# Optimized DC–DC converter based on new interleaved switched inductor capacitor for verifying high voltage gain in renewable energy applications

**DOI:** 10.1038/s41598-023-42638-5

**Published:** 2023-09-30

**Authors:** Ammar Falah Algamluoli, Xiaohua Wu, Mustafa F. Mahmood

**Affiliations:** 1 School of Automation, Northwestern Polytechnical University, Xi’an, 710072 China; 2 Electrical Engineering Technical College, Middle Technical University, Baghdad, Iraq

**Keywords:** Electrical and electronic engineering, Energy science and technology

## Abstract

This paper introduces an optimized DC–DC converter that employs a modified switched inductor-capacitor technique to achieve ultra-high voltage gain for renewable energy systems. The development is based on adding one cell of modified switched inductor (MSL1) with series diodes interleaved with the main switch in the proposed DC–DC converter. The (MSL1) with capacitor operates in resonant mode to reduce current stress across the main switch when the charge in capacitor becomes zero. This approach also reduces voltage stress across the main switch, all inductors, and diodes. Furthermore, modified switched inductors (MSL2) with an auxiliary switch and a coupled capacitor are incorporated to provide double boosting voltage and to achieve high voltage gain. Additionally, a main and auxiliary switch are integrated with modified switched capacitors (MSC) to provide ultra-high voltage gain and to reduce voltage stress across auxiliary switch. Moreover, the proposed converter exhibits a continuous input current with zero pulsating, even at very low duty cycles. The advantages of the proposed converter are high efficiency, low voltage stress, and low values of inductors and capacitors when utilizing a high switching frequency. A mathematical model for the proposed converter is developed for both continuous conduction mode and discontinuous conduction mode. In addition, the PCB design for the proposed converter is presented, and experimental tests are conducted to verify the simulation and laboratory results. The proposed converter aims to boost the voltage from 20 to 40 V to a variable output voltage between 200 and 400 V, delivering 400 watts of power with an efficiency of 96.2%.

## Introduction

Over the past few decades, attention has been drawn to the pressing issues of climate change and global warming due to greenhouse gas emissions. As a result, the necessity of reducing carbon emissions from fossil fuels has been recognized^1–8^. Sustainable energy resources, such as photovoltaic cells, FC fuel cells, and wind energy, are being utilized for electricity generation. However, these sources are characterized by a lower output voltage when compared to the voltage of the main grid connection. To increase the output voltage of photovoltaic cells, they can be connected in series. Nevertheless, a high and stable output voltage is not achieved through this method due to the shadow effect^8–29^. A suitable solution to this problem is the application of a DC–DC converter. By employing power electronic DC–DC converters with high voltage gain, the output voltage of PV panels can be enhanced^12^. However, many challenges are faced by researchers in the modification and development of new DC–DC power converters for renewable energy applications. Several conventional DC–DC converters, such as Boost, SEPIC, CUK, ZETA, and Buck Boost converters, are employed to increase low voltage to high voltage gain for various applications like Uninterruptible Power Supply, street LED lights, and medical devices. Nevertheless, these converters come with their own set of problems when utilized for achieving boosted high-voltage gain, as previously mentioned. For instance, a conventional boost converter can operate and boost low voltage up to 10 times the voltage gain^6^. However, the efficiency of traditional boost converters diminishes after high extreme duty ratios are employed. Additionally, the limited counts of inductors and capacitors render traditional converters unsuitable for ultra-high voltage gain. Furthermore, power MOSFET devices experience high voltage stress and high current stress when the converter operates at a high extreme duty ratio. Moreover, when the converter operates at high voltage with a very high duty ratio, MOSFET conduction and switching power losses significantly increase, along with the issue of reverse recovery due to diodes^5^. These converters, as mentioned earlier, have attracted the interest of many researchers aiming to achieve high voltage gains. Various topologies have been developed using coupled and non-coupled inductors, isolated and non-isolated transformers to boost voltage gain. Concerning the converters developed using coupled inductors and transformers, a dual boosting stage with a common ground coupled inductor and switched capacitor for RES was proposed in^1^, while^11^ combined two conventional CUK and SEPIC converters with coupled inductors for the same purpose. Another development is presented in^22^, which introduces a Y-source step-up converter based on three coupled inductors for boosting applications. Additionally, Nafari and Beiranvand^31^ presents an improved Z-source (ZS) converter using a coupled inductor with one core. In^32^, a modification to a DC–DC converter is proposed using a single-switch SEPIC converter with an isolated transformer and supporting Voltage Doppler (VD) cell, without using a clamped circuit. In^27^, a modified Z-source (ZS) converter with a unique ground between the energy source and load is discussed, utilizing coupled inductors and a voltage multiplier (VM) for RES applications as non-isolated. Siwakoti et al.^25^ presents a modified SEPIC converter with a coupled inductor for piezoelectric drive systems, while^28^ developed a SEPIC converter using a coupled inductor with a passive clamp circuit for high step-up gain converters. Additionally, Kushwaha et al.^24^ presents a combined conventional Cuk and SEPIC converter developed as a single stage for electric vehicle battery chargers with coupled inductors. These developed converters have been demonstrated to achieve high voltage gain but at the cost of utilizing a high number of inductors and capacitors. Moreover, coupled inductors can result in leakage inductance, leading to a very high spike voltage across the MOSFET during the off-time. This can result in the converter becoming bulky, heavy, and costly. Furthermore, using a higher number of MOSFETs and diodes leads to a significant decrease in converter efficiency, as conduction and switching losses increase. Additionally, increasing the number of turns ratio of the coupled inductor to enhance the voltage gain results in higher internal resistance, which can impair the system's efficiency and performance.

Numerous advancements have been made in non-isolated DC–DC converters to achieve high voltage transfer ratios. In^2^, a modification to the non-isolated Luo converter is proposed, employing a single switch with a hybrid switched capacitor (SC) technique. In^3^, a modified Z-source network with a double input DC–DC converter is presented to achieve a high voltage transfer gain. Additionally, Bhaskar et al.^4^ introduces an Improved Boost Converter as a multistage Switched Inductor that utilizes a polarized capacitor, while^5^ presents a single switch with a single inductor based on a new hybrid boosting converter (HBC) that employs a bipolar voltage multiplier (BVM) to enhance the voltage conversion ratio. The SEPIC structure has been enhanced by integrating it with a conventional boost using active switched capacitors (ASC) and switched inductors (ASL), as described in^10–18^, resulting in modifications in^9–34^. Furthermore, Sedaghati et al.^8^ proposed the use of two interleaved KY converters, and^16^ combined a KY converter interleaved with a conventional buck converter to form a buck-boost converter for stepping up the output voltage. While high voltage gain can be achieved by these converters, they face limitations when operating at low switching frequencies. Larger inductors and capacitors are necessitated by low switching frequencies, increasing the internal resistance of MOSFETs, leading to higher power losses and reduced efficiency. Additionally, conduction and switching losses can be elevated by a high number of diodes and MOSFETs, further reducing efficiency. The impact on voltage gain values is also exacerbated by the use of diodes with a high forward voltage, contributing to converter performance issues. Other topologies, such as the conventional ZS impedance network converter with (SC) proposed in^7^, and the modified traditional buck-boost converter with an additional switch in^14^, have also been suggested. In^15^, a traditional boost converter was developed by adding a switched network with two conventional four-quadrant switches, while in^19^, a SC with a diode was added. In^17–30^, a traditional SEPIC converter is combined with a conventional boost converter and with (n) number of voltage multipliers in^26^, and an active (SL) combined with passive inductors in^12^, to achieve high step-up voltage gain. Two traditional SEPIC converters are combined in^20^^,^^21^, a converter with a dual-switch and (SC) is proposed to attain a high voltage gain. Maalandish et al.^23^ presents two modified three-phase interleaved boost converters, each with two (SC) in every phase, while^33^ describes a conventional SEPIC converter with a combination of inductors, capacitors, and diodes. These non-isolated converters, designed for use in photovoltaic (PV) applications, have the potential to achieve high conversion ratios. However, as mentioned earlier, lower voltage gain is exhibited by these converters with pulsating input current at low duty cycles. Additionally, complex control circuits are required for the power MOSFETs, and the input current can follow more than one path during the on and off states. Moreover, power MOSFETs and diodes are subjected to high voltage and current stresses during the on and off states. Furthermore, high conduction and switching losses are incurred by these converters at high extreme duty cycles in achieving high voltage gain. Additionally, a high number of switches is required to achieve high voltage gain.

**Figure 1:**
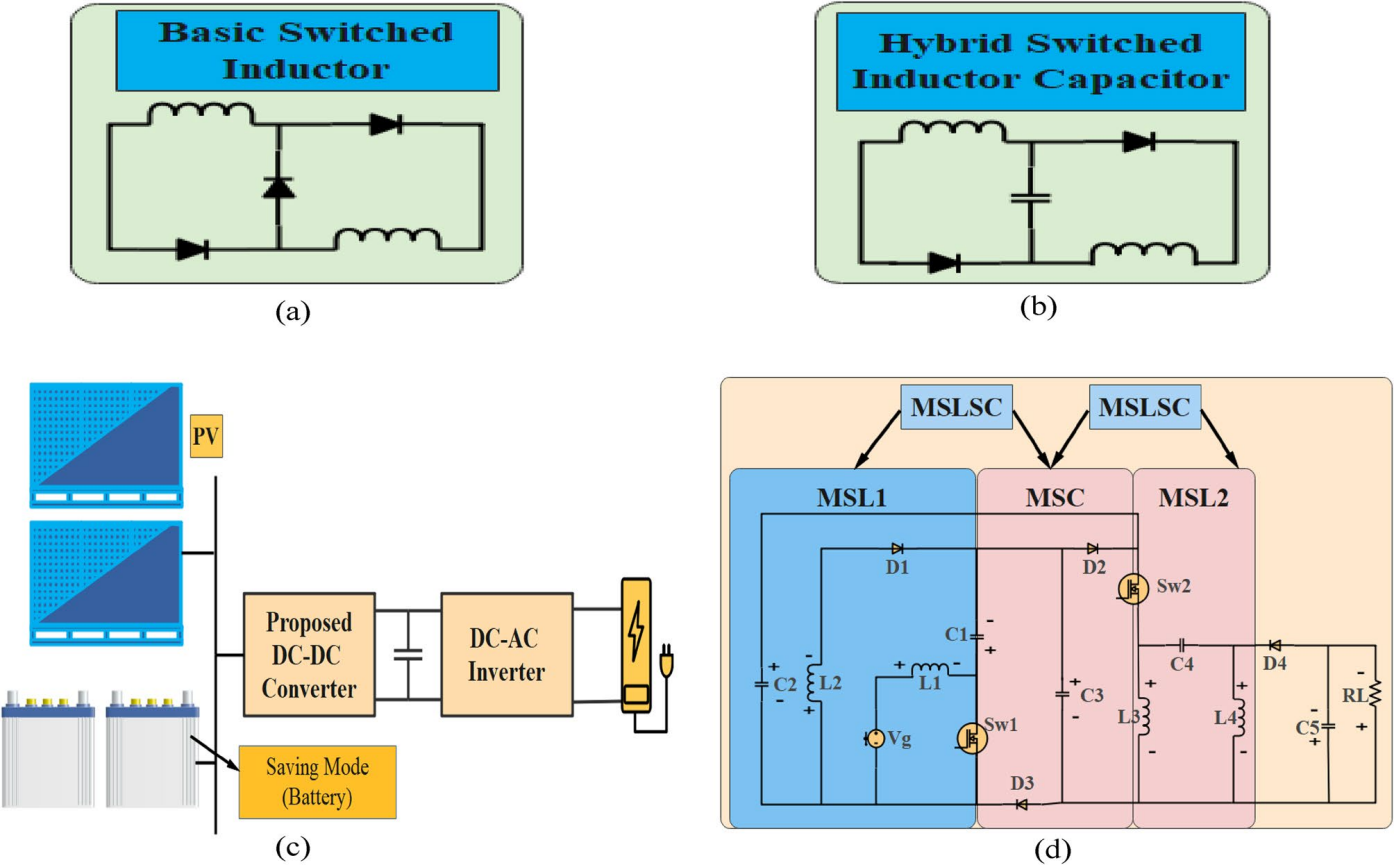
(**a**) Connection of a basic switched inductor, (**b**) a hybrid connection both a switched inductor and a capacitor (**c**), the proposed converter connects with PV Panels and Batteries, designed for Energy Saving Mode Applications (**d**). The structure of proposed DC–DC converter.

This paper optimized a DC–DC converter that utilizes a modified (MSLSC) technique to achieve ultra-high voltage gain for renewable energy sources (RES). The development is based on adding one cell of (MSL1) (L_2_ and C_1_) with series diodes interleaved with the main switch in the proposed DC–DC converter, as shown in Fig. 1b. This MSL1 with capacitor operates in resonant mode to reduce current stress across the main switch when charge in capacitor C1 becomes zero. In addition, this approach also reduces voltage stress across the main, auxiliary switches and diodes (D_1_, D_2_, and D_3_). Furthermore, (MSL2) with an auxiliary switch and a coupled capacitor are incorporated to provide double boosting voltage and achieve high voltage gain. Additionally, main and auxiliary switches are integrated with (MSC) to provide ultra-high voltage gain. Moreover, the proposed converter exhibits a continuous input current with zero pulsating at a very low duty cycle. In addition, D_2_ and D_3_ operate in zero voltage switching (ZVS), as shown in Fig. 3c, at minimum input voltage and at high load current. The proposed DC DC converter operate at DCM and CCM with zero pulsating input current at low duty cycle. In addition, the proposed DC–DC converter can supply variable high-output voltage between 200 and 400 V, making it more suitable for a wide range of applications. Furthermore, a dual PI controller is designed for the proposed converter to maintain a fixed output voltage under variable load and input voltage conditions.

## Structure and operation principle of the proposed converter

The structure of the proposed converter consists of four main low-value inductors, five capacitors, four diodes, and two power MOSFET switches (main and auxiliary) as shown in Fig. 1d. Where the main switch is Sw_1_ and auxiliary switch is Sw_2_. The proposed DC–DC converter has been modified to incorporate a basic switched inductor and a hybrid switched inductor-capacitor, as illustrated in Fig. 1a,b. The interconnection between the suggested converter, photovoltaic panels, and a battery, specifically for applications related to energy-saving modes is shown in Fig. 1c. The proposed DC–DC converter has been developed to achieve an ultra-high voltage gain, capable of boosting a low input voltage range of 20–40 V to a variable output voltage between 200 and 400 V under a 400 W load, making the system more suitable for a wide range of applications. The proposed DC–DC converter offers several advantages over previous converters. Firstly, it eliminates the need for isolated coupled inductors and transformers to step up the voltage, resulting in reduced values of inductors and capacitors at high switching frequencies. This enhances the efficiency of the proposed converter. Moreover, the structure of the converter is simple and easy to implement. The proposed converter is also highly reliable for renewable energy system (RES) applications as it ensures no pulsating input current at low duty cycle. In addition, L_2_ and C_1_ operate at resonant mode, which reduces the current stress through Sw_1_ when the charge in capacitor C_1_ becomes zero at (D-2m), as shown in Fig. 11b,f. In addition, D_2_ and D_3_ operate in (ZVS) as shown in Fig. 13c at minimum input voltage and at high load current. Furthermore, the MOSFET switches and diodes experience very low voltage stress. Moreover, the voltage stress across inductors is also reduced when L_2_ and C_1_ operate at resonant mode. Additionally, the proposed converter achieves a higher voltage gain at high switching frequency than previous DC–DC converters. It can accommodate a wide range of duty cycles for high-power applications and boost low input voltage to high output voltage at very low duty cycles without the use of coupled inductors and transformers. The PWM generator of the MOSFET switches is simple, and both MOSFET devices turn on and off at the same time. The converter is characterized by its small size, low cost, and high efficiency, achieved by using capacitors and inductors with very small values.

## Operation of the proposed converter

The proposed converter is illustrated in Fig. 1d and is capable of operating in two modes. The first mode, known as DCM, has two conditions: DCMC1 and DCMC2. DCMC1 occurs at the maximum input voltage with four states of operation during one cycle at very low duty ratios. DCMC2 occurs when the converter operates at the minimum input voltage with a duty ratio of approximately 33%, as shown in Fig.  4a,b. The proposed converter can also function in CCM at high load currents when duty ratio is above 70%.

### The proposed converter operation at DCMC1

The proposed converter can operate in DCMC1 with five modes of operation, as illustrated in Fig. 3a, which presents the waveform of the proposed converter in DCMC1. This mode appears in light load applications with a duty cycle below 30%, as shown in Fig. 4a, b. In this mode, the input current remains in (CCM) at both low and high duty ratios. Conversely, the current through L3 and L4 operates in (DCM).

**Figure 2:**
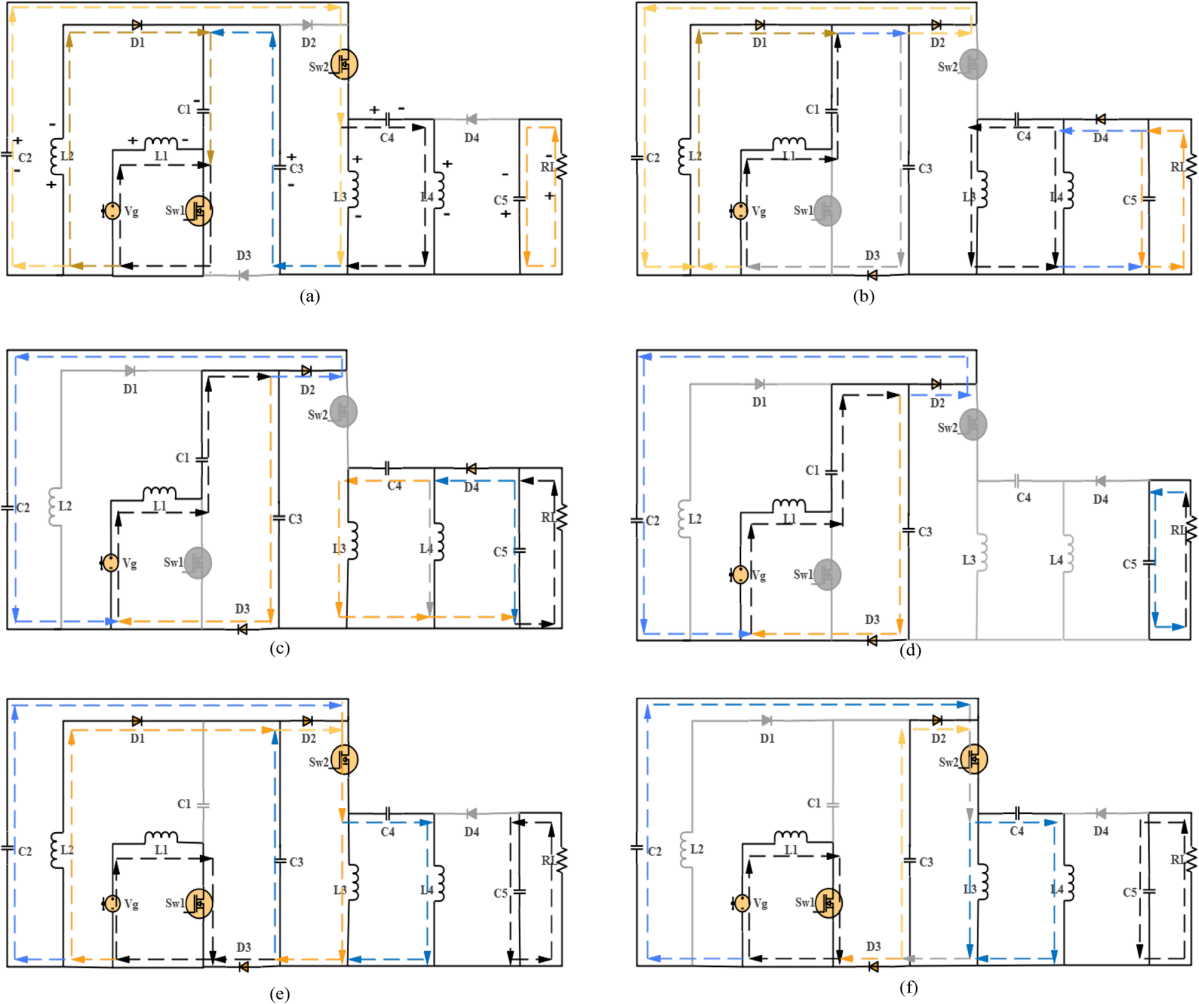
(**a**) Mode 1 DCMC1, Mode 1 DCMC2, (**b**) Mode 2 DCMC1, (**c**) Mode 3 DCMC1, Mode 4 DCMC2, Mode 4 CCM, (**d**) Mode 4 DCMC1, Mode 5 DCMC2, Mode 3 DCMC1, (**e**) Mode 1 DCMC2, Mode 2 DCMC2, Mode 2 CCM , (**f**) Mode 3 DCMC2,Mode 2 DCMC2, Mode 2 CCM, Mode 3 CCM.

*Mode 1* [0–t0] Both MOSFETs S_w1_ and Sw_2_ are simultaneously switched on, causing diodes D_2_, D_3_, and D_4_ to be turned off. Energy is linearly charged from the input source Vg by L_1_ since it is in series with it. L_2_ is charged from C_1_ through Sw_1_. In this mode, D_1_ is switched on, and capacitors C_2_ and C_3_ are connected in series. They are employed to charge L_3_ through Sw_2_, while L_4_ is charged from C_4_. Energy is provided to the load by C_5_. The converter for this mode is illustrated in Fig. 2a. Below are the voltage and current equations for the various components in this mode:

1 $$\left. \begin{gathered} VL_{1} = V_{g} \hfill \\ VL_{2} = Vc_{1} \hfill \\ VL_{3} = Vc_{1} + Vc_{2} + Vc_{3} \hfill \\ VL_{4} = Vc_{1} + Vc_{2} + Vc_{3} - Vc_{4} \hfill \\ Vc_{5} = V_{o} \hfill \\ \end{gathered} \right\}$$

2 $$iL_{1} + iL_{2} = Isw_{1}$$

3 $$\left. \begin{gathered} Ic_{2} = Ic_{3} = Isw_{2} = iL_{3} \hfill \\ iL_{4} = Ic_{4} \hfill \\ \end{gathered} \right\}$$

4 $$ID_{1} = iL_{2} = IC_{1}$$

5 $$IC_{5} = I_{o}$$

6 $$\left. \begin{gathered} iL_{1} = \frac{{V_{g} }}{{L_{1} }} \hfill \\ iL_{2} = \frac{{Vc_{1} }}{{L_{2} }} \hfill \\ iL_{3} = \frac{{Vc_{2} + Vc_{3} + Vc_{1} }}{{L_{3} }} \hfill \\ iL_{4} = \frac{{Vc_{2} + Vc_{3} + Vc_{1} - Vc_{4} }}{{L_{4} }} \hfill \\ I_{o} = \frac{{V_{o} }}{RL} \hfill \\ \end{gathered} \right\}$$

where VL is the voltage across the inductor, iL is the current through the inductor, Ic is the current through the capacitor, Io is the output current, ID is the diode current, Vg is the input voltage source, Vo is the output voltage of the proposed converter, Vc is the voltage across the capacitor, and RL is the resistive load.

*Mode 2* [t0–t1] the power MOSFETs are in the off state as zero gate voltage is provided to them by the Pulse generator to keep them off. Both diodes D_2_ and D_3_ are in the on state, and D_4_ is also in the on state during this mode. L_1_ will discharge its energy to C_1_ while charging of C_2_ and C_3_ occurs in a nonlinear manner. L_2_ will begin to discharge its power to C_2_ and C_3_. D_1_ remains in the on state in this mode due to the discharging current of L_2_ through it. C_2_ and C_3_ will start storing a significant amount of energy from L_1_ and L_2_. C_5_ will receive a large amount of energy from L_3_ and L_4_ during the discharging time, allowing high power to be supplied to the load. The proposed converter for this mode is depicted in Fig. 2b. Here are the voltage and current equations for the various components in this state:

7 $$\left. \begin{gathered} VL_{1} = V_{g} - Vc_{1} - Vc_{2} \hfill \\ VL_{2} = - Vc_{2} \hfill \\ VL_{3} = Vc_{4} + VL_{4} \hfill \\ VL_{4} = - Vc_{5} \hfill \\ Vc_{5} = V_{o} \hfill \\ \end{gathered} \right\}$$

8 $$\left. \begin{gathered} iL_{1} = (\frac{Vg}{{L_{1} }} - \frac{{Vc_{1} }}{{L_{1} }} - \frac{{Vc_{2} }}{{L_{1} }}) \hfill \\ iL_{2} = \frac{{ - Vc_{2} }}{{L_{2} }} \hfill \\ iL_{3} = \frac{{Vc_{4} + VL_{4} }}{{L_{3} }} \hfill \\ iL_{4} = \frac{{ - V_{o} }}{{L_{4} }} \hfill \\ I_{o} = \frac{{V_{o} }}{RL} \hfill \\ \end{gathered} \right\}$$

9 $$iL_{1} + iL_{2} = ID_{2} + ID_{3} = Ic_{2} + Ic_{3}$$

10 $$iL_{3} + iL_{4} = ID_{4}$$

11 $$ID_{1} = iL_{2}$$

12 $$iL_{1} = Ic_{1}$$

*Mode 3* [t1–t2]: The power MOSFETs are still in the off state, and diodes D_2_ and D_3_ remain in the on state. The energy of L_1_ is discharged nonlinearly due to resonance with C_1_, resulting in energy transfer to C_1_ to minimize input current ripple and to charge C_2_ and C_3_. During the time (D + m1), L_2_ is not charged and reaches zero, resulting in ID_1_ being zero. Energy is continued to be received by C_2_ and C_3_ only from L_1_. Energy continues to be supplied to C_5_ from L_3_ and L_4_ during the discharging period, while high power is still received by the load from C_5_. The proposed converter for this mode is shown in Fig. 2c. The voltage equations remain the same as in the previous mode, and the current equations for this state are as follows:

13 $$Ic_{5} = Io = iL_{3} + iL_{4}$$

14 $$iL_{1} = ID_{2} + ID_{3}$$

15 $$ID_{1} = iL_{2} = 0$$

16 $$Ic_{2} + Ic_{3} = iL_{1}$$

**Figure 3:**
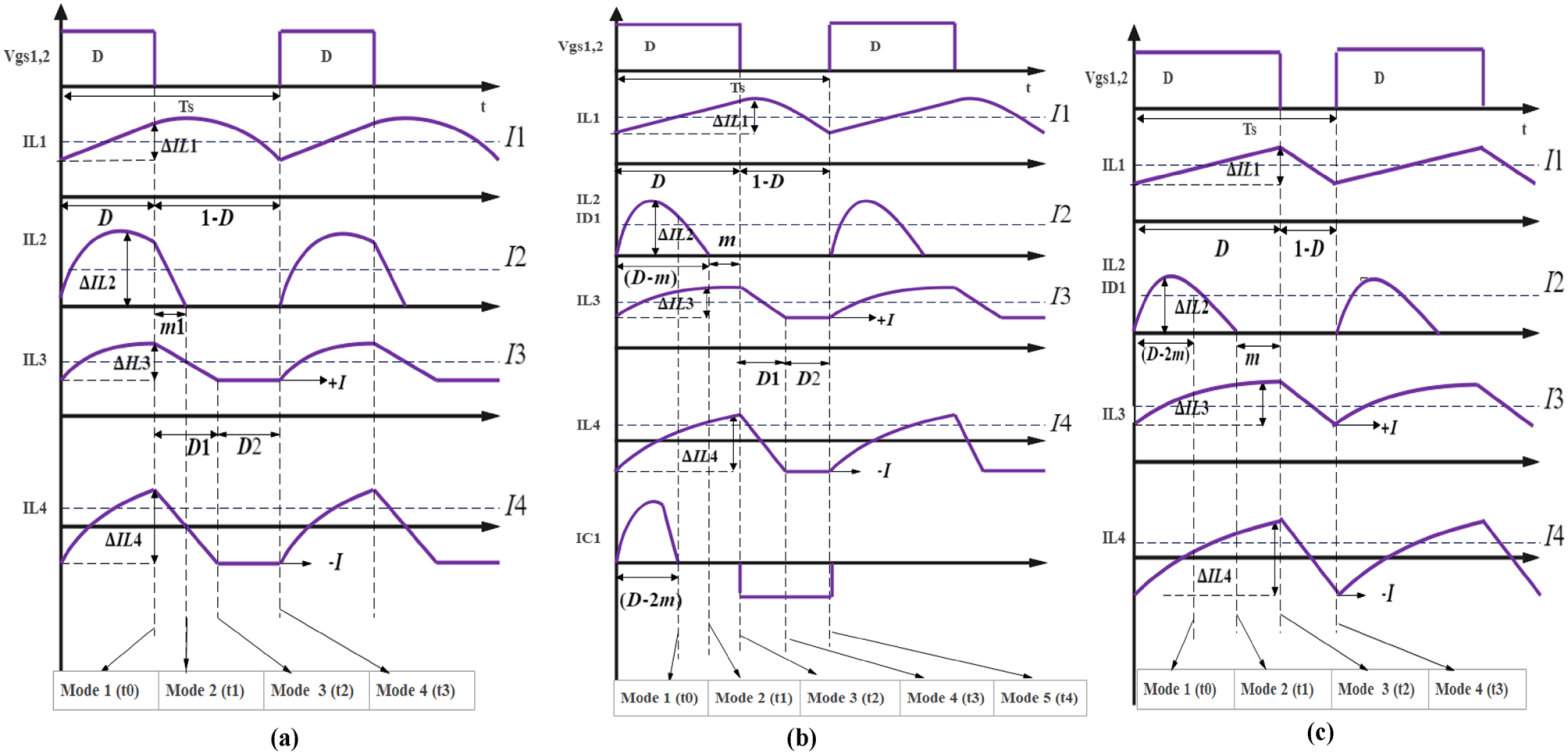
(**a**) Time-domain waveforms for the proposed DC–DC converter operating in DCMC1. (**b**) Time domain waveforms for the proposed DC–DC converter operating in DCMC 2. (**c**) Time domain waveforms for the proposed DC–DC converter operating in CCM.

*Mode 4* [t2–t3]: The power MOSFETs are still in the off state, and only D_2_ and D_3_ remain on. In this mode, D_4_ is turned off, while D_1_ remains off. The energy stored in L_1_ is discharged through C_1_ to charge C_2_ and C_3_, which store a large amount of energy for the next gate pulse to supply a substantial amount of energy to the load. L_4_ will have the same current values as L_3_ but in opposite directions (iL_3_ = − iL_4_). C_5_ supplies high power to the load. The proposed converter for this mode is shown in Fig. 2d. The waveforms for these four operational modes are illustrated in Fig. 3a.

### Operation of the proposed converter in DCMC2

When Vg is decreased to its minimum value, the proposed converter can operate in DCMC2 with five modes of operation, as illustrated in Fig. 3b, which presents the waveform of the proposed converter in DCMC2. In this mode, the input current remains in CCM at both low and high duty ratios, while C_1_ operates in a resonant mode with L_2_. Conversely, the current through L_3_ and L_4_ operates in DCM. This approach effectively reduces the voltage stress across the auxiliary switch, thereby decreasing the losses in the proposed converter.

*Mode 1* [0–t0]: Both Sw_1_ and Sw_2_ are simultaneously switched on, resulting in the switching off of diodes D_2_, D_3_, and D_4_. L_1_ accumulates energy linearly from the input source Vg. L_2_ accumulates energy from C_1_ through Sw_1_ until (D-2m), at which point the charge in C_1_ becomes zero. D_1_ is switched on, and the capacitors currents C_2_ and C_3_ are utilized to charge L_3_ through Sw_2_, while L_4_ charges from C_4_. C_5_ supplies energy to the load. The proposed converter for this mode is illustrated in Fig. 2a,e.

*Mode 2* [t0–t1]: The power MOSFETs remain in the on state. Both diodes, D_2_ and D_3_, turn on during this state because the charge in C_1_ becomes zero at (D-2 m), as shown in Fig. 3b. Consequently, L_2_ begins to discharge energy through D_2_ and D_3_ to charge L_3_ and L_4_. This reduces the current stress through Sw_1_, as depicted in Fig. 11b. The current through Sw_1_ is solely sourced from L_1_. D_4_ remains off, and L_1_ continues to accumulate energy from the input voltage source. In this mode, D_1_ experiences very low voltage stress, implying that L_2_ acts as an open circuit during the time (D-m). Furthermore, in this mode, D_2_ and D_3_ experience (ZVS) across them from ((D-2m) < t < (D-m)). After this time, both of them work as a path to pass the current from L_2_. L_3_ and L_4_ continue to charge from C_2_, C_3_ and L_2_. L_2_'s charge becomes zero at (D-m). C_5_ still supplies power to the load. The proposed converter for this mode is shown in Fig. 2e,f. The current equations for this state are provided below.

17 $$\left. \begin{gathered} iL_{2} = \frac{{Vc_{1} }}{{L_{2} }}(D - 2m) \hfill \\ iL_{2} = \frac{{( - Vc_{2} )}}{{L_{2} }}\;from\;(D - 2m) < t < (D - m) \hfill \\ iL_{3} = \frac{{Vc_{2} + Vc_{3} }}{{L_{3} }},\;from\; \, D < t < (D - 2m) \hfill \\ iL_{3} = \frac{{Vc_{3} }}{{L_{3} }},\;from \, \;(D - 2m) < t < D \hfill \\ \end{gathered} \right\}$$

18 $$\left. \begin{gathered} Isw_{1} = iL_{1} + iL_{2} ,\;from\;0 < t < (D - 2m) \hfill \\ Isw_{1} = iL_{1} (only)\;from\;(D - 2m) < t < D \hfill \\ Isw_{2} = Ic_{2} = Ic_{3} ,\;from\;0 < t < (D - 2m) \hfill \\ Isw_{2} = Ic_{2} + Ic_{3} ,\;from\;(D - 2m) < t < D \hfill \\ \end{gathered} \right\}$$

*Mode 3* [t1–t2] The Power MOSFETs remain in the "on" state, and both diodes D_2_ and D_3_ are still turned on. L_2_ is treated as an open circuit, with D_1_ is switched off. L_1_ continues to charge from the input source, and L_3_ and L_4_ will continue to accumulate current from C_2_ and C_3_ through Sw_2_. C_5_ will begin accumulating a larger amount of energy from L_3_ and L_4_ through D_4_, and this energy will be used to supply power to the load. The proposed converter for this mode is depicted in Fig. 2f. The current equations for this mode are presented in Eq. (18).

*Mode 4* [t2–t3] The Power MOSFETs are both turned off, and D_2_ and D_3_ remain in the "on" state, while D_4_ is switched on. L_2_ is treated as an open circuit in this mode, and L_1_ is discharging energy to charge C_1_, C_2_, and C_3_ while storing a significant amount of energy to supply to the load. C_5_ continues to receive energy from L_3_ and L_4_ during the discharging period (D1), while the load continues to receive high power from C_5_. The proposed converter for this mode is illustrated in Fig. 2c.

*Mode 5* [t3–t4]: Both MOSFETs remain in the off state, and D_2_ and D_3_ are still on. D_4_ is now turned off. During this period (D2), L_3_ will carry the same current as L_4_ but in opposite directions: iL_3_ = − iL_4_. The load will receive a high amount of energy from C_5_. The proposed converter for this mode is depicted in Fig. 2d.

### Operation of the proposed converter in CCM

**Figure 4:**
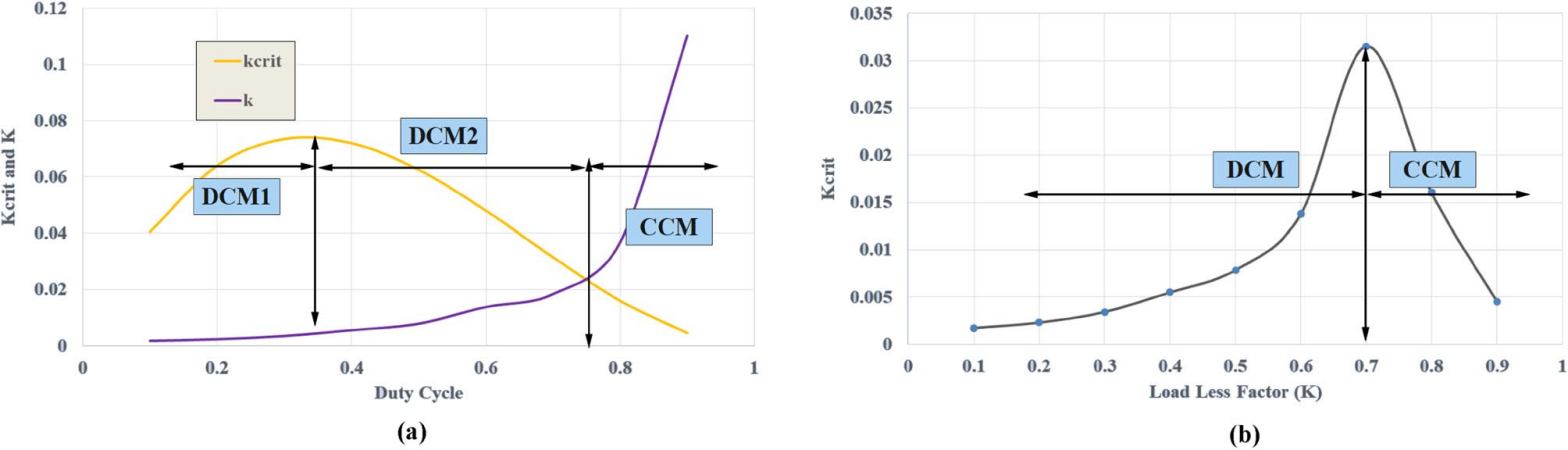
Dynamic response of the proposed converter (**a**) Kcrit and K versus duty cycle, (**b**) load less factor (K) versus (Kcrit).

This mode is activated when the load current surpasses a 70% duty cycle, as depicted in Fig. 4, and when the load factor (k) exceeds the critical value of K (Kcrit). In this mode, the proposed converter maintains the input current in CCM, with L_2_ still operating in resonance mode with C_1_, and the current through L_3_ and L_4_ also remains in CCM. Furthermore, the voltage transfer ratio in this mode experiences a significant increase. When the proposed converter operates in CCM, it exhibits four distinct modes of operation. The time-domain waveforms for the proposed DC–DC converter operating in CCM are also illustrated in Fig. 3c.

*Mode 1* [0–t0]: same as for Mode 1 of DCMC2

*Mode 2* [t0–t1]: same as for Mode 2 of DCMC2

*Mode 3* [t0–t1]: same as for Mode 3 of DCMC2

*Mode 4* [t2–t3]: Both MOSFETs Sw_1_ and Sw_2_ are in the off state, D_2_ and D_3_ remain on. L_1_ will discharge energy linearly due to the short discharging time required to charge C_1_, C_2_, and C_3_ with a large amount of power. L_2_ is an open circuit with no current, and D_1_ remains off. Both capacitors C_2_ and C_3_ accumulate a significant amount of energy from L_1_, while C_4_ starts to store a large amount of energy from L_3_ and L_4_. The current waveform of L_3_ and L_4_ will be the same but in opposite directions, without a constant current (I), and the load receives a substantial power supply from C_5_ through D_4_. The proposed converter for this mode is shown in Fig. 2c.

## Voltage transfer gain calculation

The voltage transfer gain of the proposed converter is calculated under two conditions when the converter operates in DCM and CCM.

### Voltage transfer gain calculation at DCMC1

To derive the voltage gain equation for the proposed converter operating in DCMC1, the volt-second balance equation is applied to L_1_, L_2_, L_3_, and L_4_ using Eqs. (1) and (7). This process yields Eqs. (19) and (20), which can be solved to obtain Eqs. (22) and (24). Additionally, during the steady state in DCMC1 at low duty cycle, the average voltage across C_1_ can be determined using Eq. (21). Equation (22) establishes the relationship between C_2_ and the input voltage source. In this context, (m1) represents the discharging time of L_2_, and its values can be obtained from Eq. (23), which is a function of (D, Vg, Vc_2_, and Vc_3_). (D1) denotes the discharging time of L_3_ and L_4_, and it can be calculated using Eq. (24).

19 $$\frac{1}{Ts}\left( {\int_{0}^{DTs} {(V_{g} + Vc_{1} )dt} + \int_{D}^{Ts} {(V_{g} - Vc_{1} - Vc_{2} )} dt + \int_{DTs}^{m1Ts} {( - Vc_{2} } )dt} \right) = 0$$

20 $$\frac{1}{Ts}\left( {\int_{0}^{DTs} {2(Vc_{2} + Vc_{3} + Vc_{1} ) - Vc_{4} dt} + \int_{D}^{D1Ts} {( - 2Vo)} dt} \right) = 0$$

21 $$Vc_{1} = \frac{Vg}{{(1 - D)}} - Vc_{2}$$

22 $$Vc_{2} = \frac{{V_{g} D}}{(1 - D)(D + m1)}$$

23 $$m1 = \frac{{V_{g} D}}{{Vc_{2} (1 - D)}} - D$$

24 $$D1 = \frac{{D(Vc_{2} + Vc_{3} + Vc_{1} )}}{{V_{o} }}$$

25 $$\left. \begin{gathered} < I1 > = \frac{{V_{g} }}{{2L_{1} }}DTs \hfill \\ < I2 > = \frac{{V_{g} D^{2} (V_{g} - (1 - D)Vc_{2} )}}{{2fsL_{2} (1 - D)^{2} Vc_{2} }} \hfill \\ \end{gathered} \right\}$$

26 $$\left. \begin{gathered} iL_{1} peak = \frac{{V_{g} DTs}}{{L_{1} }} \hfill \\ iL_{{2 Peak}} = \frac{{(V_{g} - (1 - D)Vc_{2} )DTs}}{{L_{2} (1 - D)}} \hfill \\ \end{gathered} \right\}$$

Equations (25) and (26) represent the average and peak current through inductors L_1_ and L_2_, respectively. Equation (27) provides the average inductor current in L_3_ and L_4_. Meanwhile, Eqs. (28) and (29) deal with the peak inductor current in L_3_ and L_4_, respectively. To find the output current, we can calculate it by adding the currents from Eqs. (27), as demonstrated in Eq. (30). In this context, (LE) represents the equivalent inductors of L_3_ and L_4_, as shown in Eq. (31).

27 $$\left. \begin{gathered} < I3 > = \frac{{(Vc_{2} + Vc_{3} + Vc_{1} )}}{{2L_{3} }}DTs(D + D1) + I \hfill \\ < I4 > = \frac{{(Vc_{2} + Vc_{3} + Vc_{1} - Vc_{4} )}}{{2L_{4} }}DTs(D + D1) - I \hfill \\ \end{gathered} \right\}$$

28 $$iL_{3peak} = \frac{{(Vc_{2} + Vc_{3} + Vc_{1} )}}{{L_{3} }}DTs$$

29 $$iL_{4peak} = \frac{{(Vc_{2} + Vc_{3} + Vc_{1} - Vc_{4} )}}{{L_{4} }}DTs$$

30 $$I_{o} = \frac{{(Vc_{2} + Vc_{3} + Vc_{1} )DD1}}{FsLE}$$

31 $$LE = \frac{{L_{3} L_{4} }}{{L_{3} + L_{4} }}$$

32 $$Vo = \frac{{(Vc_{2} + Vc_{3} )^{2} D^{2} }}{LEIoFs}$$

Equation (32) represents the output voltage of the proposed converter. The voltage gain equation for the proposed converter in DCMC1 can be obtained through Eq. (33), and solving this equation helps determine the load factor (K). Consequently, the voltage gain equation for the proposed converter as a function of K can be derived in Eq. (34). To find the critical load factor (Kcrit), we can use Eq. (35). The boundary condition of the proposed converter between CCM and DCM at Kcrit is shown in Eq. (36).

33 $$VG(DCMC1) = \frac{{2VgD^{4} TsRL}}{{LEV_{o} (1 - D)^{2} (D + m1)^{2} }}$$

34 $$\left. \begin{gathered} VG(DCMC1) = \frac{{D^{2} }}{(1 - D)(D + m1)}\sqrt{\frac{2}{k}} \hfill \\ K \, = \frac{2LE}{{TsR_{L} }} \hfill \\ \end{gathered} \right\}$$

35 $$k_{crit} = \frac{{(1 - D)^{2} D}}{2}$$

36 $$K_{crit} = \left\{ {\begin{array}{*{20}c} {{\text{if}}} & {{\text{Kcrit > K}}\;{\text{Proposed}}\;{\text{Converter}}\;{\text{work}}\;{\text{In}}\;{\text{DCM}}} \\ {{\text{if}}} & {{\text{Kcrit}}\;{\text < {K}}\;{\text{Proposed}}\;{\text{Converter}}\;{\text{work}}\;{\text{In}}\;{\text{CCM}}} \\ \end{array} } \right.$$

### Voltage transfer gain calculation at DCMC2

To derive the voltage gain equation for the proposed converter when it operates in DCMC2, volt-second balance equations are applied to L_1_, L_2_, L_3_, and L_4_ using Eqs. (17) and (7), which results in Eq. (37). Solving this equation leads to the results shown in Eqs. (38).

37 $$\left. \begin{gathered} \frac{1}{Ts}\left( {\int_{0}^{DTs} {(V_{g} )dt} + \int_{(D - 2m)T}^{(D - m)Ts} {( - Vc_{2} )dt} } \right) \hfill \\ \quad + \int_{0}^{(D - 2m)Ts} {(Vc_{1} )dt} + \int_{D}^{Ts} {(V_{g} - Vc_{1} - Vc_{2} )dt} ) \hfill \\ \quad + \frac{1}{Ts}(\int_{0}^{(D - 2m)Ts} {2(Vc_{2} + Vc_{3} )dt} + \int_{(D - 2m)}^{(D - m)Ts} {2(Vc_{3} )dt} \hfill \\ \quad + \int_{D}^{D1Ts} {( - 2Vo)} dt = 0 \hfill \\ \end{gathered} \right\}$$

38 $$\left. \begin{gathered} D(V_{g} ) - m(Vc_{2} ) + (1 - D)(V_{g} - Vc_{1} - Vc_{2} ) + (D - 2m)(Vc_{1} ) \hfill \\ 2(D - 2m)(Vc_{2} + Vc_{3} ) + 4mVc_{3} - 2D1Vo = 0 \hfill \\ \end{gathered} \right\}$$

39 $$m = D - \frac{{\sqrt {L_{2} C_{1} } }}{Ts}$$

40 $$Vc_{2} = \frac{Vg}{{(1 - D)}}$$

41 $$D1 = \frac{{D(Vc_{2} + Vc_{3} )}}{{V_{o} }}$$

42 $$VG(DCMC2) = \frac{{2VgD^{2} TsRL}}{{LEV_{o} (1 - D)^{2} }}$$

The value of (m) can be determined using Eq. (39), which depends on the values of L_2_ and C_1_. This approach reduces the current stress across the main switch as the load current increases. Additionally, it lowers the voltage stress across both the main and auxiliary switches, resulting in a reduction in voltage stress on all switched inductors, and diodes. When the charge in C_1_ reaches zero at (D-2m), the two diodes D_2_ and D_3_ will operate at ZVS, as shown in Fig. 13c. Equation (40) describes the voltage across C_2_. The discharging time of L_3_ and L_4_, denoted as (D1), can be determined from Eq. (41) after solving Eq. (38). Finally, the voltage gain equation of the proposed converter in DCMC2 is provided in Eq. (42).

### Voltage transfer gain calculation at CCM

By applying the volt-second balance equation to L_1_, L_2_, L_3_, and L_4_ in Eq. (43) at different times, the results can be found in (44) and (45) by substituting the value of Vc_1_ from Eq. (21) into Eq. (44). The voltage transfer gain of the proposed DC–DC converter when operating in CCM is given by Eq. (46). It can be observed that the voltage gain ratio equations of the proposed converter in DCM and CCM exhibit higher voltage gain ratios than those of previous DC–DC converters, as shown in Table 2.

43 $$\frac{1}{Ts}\left( {\int_{0}^{DTs} {(V_{g} )dt} + \int_{D}^{Ts} {(V_{g} - Vc_{1} - Vc_{2} )} dt} \right) = 0$$

44 $$D(V_{g} ) + (1 - D)(V_{g} - Vc_{1} - Vc_{2} - Vc_{3} ) = 0$$

45 $$D(Vc_{2} + Vc_{3} ) = (1 - D)V_{o}$$

46 $$VGCCM = \frac{{V_{o} }}{{V_{g} }} = \frac{2D}{{(1 - D)^{2} }}$$

## Voltage stress across power MOSFETs and diodes

In this section, the voltage stresses across the four power diodes and the voltage stresses across the power MOSFETs main and auxiliary are calculated both in DCM and CCM.

### Voltage across power MOSFETs and diodes in DCM

From Eq. (47), it can be determined that the voltage across D_1_ depends on the average voltage across C_2_, which is a very small value. Additionally, Eq. (48) provides information about the voltage across diodes D_2_ and D_3_ when the converter is operated in DCM, which is also very small and depends on the input voltage. Equation (49) allows us to calculate the voltage across MOSFET Sw_1_, which is equal to the input voltage as an average voltage. Equations (50) and (51) enable us to calculate the voltage across power MOSFET Sw_2_ and the voltage across D_4_, respectively. It can be seen that the voltage stress across diodes and switches is significantly reduced when the proposed converter is operated in DCMC1.

47 $$VD_{1} = \sqrt {\frac{{LEV_{o}^{2} }}{{RLD^{2} Ts}}}$$

48 $$VD_{2} = \frac{{V_{g} }}{(1 - D)} = VD_{3}$$

49 $$Vs_{1} = V_{g} \, \;average \, \;voltage\; \, across\;{\text{ Sw}}_{{1}}$$

50 $$Vs_{2} = \sqrt {\frac{{LEV_{o}^{2} }}{{RLD^{2} Ts}}}$$

51 $$VD_{4} = V_{o}$$

### Voltage across power MOSFETs and diodes in CCM

Equation (52) allows us to determine the voltage across D_1_ in CCM. During the time period (D-m), the voltage across D_1_ is reduced due to the resonant mode between L_2_ and C_1_. Additionally, inductor L_2_ remains uncharged and functions as an open circuit during this period. By utilizing Eq. (53), the voltage stress across Sw_1_ can be determined, and Eq. (54) can be used to find the voltage across diodes D_2_ and D_3_ in CCM. The voltage stress on power MOSFETs and power diodes is significantly reduced, resulting in decreased losses for the proposed DC–DC converter.

52 $$VD_{1} = \frac{{V_{g} }}{2(1 - D)}$$

53 $$VD_{2} = \frac{{(1 - D)V_{o} }}{D} = VD_{3}$$

54 $$Vs_{1} = \frac{{V_{g} }}{(1 - D)}$$

55 $$Vs_{2} = \frac{{V_{o} (1 - D)}}{2D}$$

56 $$VD_{4} = \frac{{2V_{g} D}}{{(1 - D)^{2} }}$$

Furthermore, the voltage stress across D_2_ and D_3_ decreases, as both diodes operate with (ZVS) across them when one cell switched capacitor charge in C1 becomes zero from (D-2m < t < D), as shown in Fig.  11f. At the time (D-2m), the inductor current L_2_ flows through diodes D_2_ and D_3_, as depicted in Fig. 11c. Upon observing Fig. 4a,b, it becomes evident that the proposed converter operates in DCMC1 when the duty cycle is below 30%, provided that Kcrit (a critical factor) is lower than the load factor K. When the input voltage decreases to its minimum value, the converter operates in DCMC2 with a duty cycle above 30%. However, as the load increases and K exceeds Kcrit, the suggested converter can operate in CCM, as shown in Fig. 4, with a duty ratio exceeding 70%.

## Proposed converter design: component selection strategies

In this section, components for a 400 W prototype of the proposed DC–DC converter have been designed to validate the experimental results. The suggested converter comprises four inductors with very small values, five capacitors with low values, two power MOSFETs with low on-state resistance (Ron), and a simple gate drive circuit, along with four power diodes. The components have been designed to achieve a high voltage transfer gain, and their specifications for the 400 W model can be found in Table 1. To design the capacitors and inductors of the converter, Eq. (57) can be utilized to calculate the value of L_1_ with low ripple input current. Ripple of input current ($\Delta iL_{1}$) can be found in Eq. (57). The resonant mode between C_1_ and L_2_ can be employed to find the value of L_2_ from Eq. (58). The values of inductors L_3_ and L_4_ can be obtained from Eq. (59), where L_3_ and L_4_ are in parallel connection. The values of C_2_ and C_3_ with very low ripple voltage can be derived from Eq. (60), and the value of C_4_ can be determined using Eq. (61). Finally, the value of C_5_ can be computed from Eq. (62) to achieve very low output voltage ripple.

57 $$\left. \begin{gathered} L_{1} = \frac{{V_{g} D}}{{Fs\Delta iL_{1} }} \hfill \\ \Delta iL_{1} = (10\% to25\% )\frac{Vo}{{Vg}}Io_{\max } \hfill \\ \end{gathered} \right\}$$

58 $$\left. \begin{gathered} L_{2} = \frac{{\Delta VRL(1 - D)^{2} }}{{8VoD^{2} \pi^{2} fS}} \hfill \\ C_{1} = \frac{{2V_{out} D^{2} }}{{\Delta VR_{L} Fs(1 - D)^{2} }} \hfill \\ \end{gathered} \right\}$$

59 $$\left. \begin{gathered} L_{3} \ge \frac{{V_{g} (1 - D)R_{L} }}{{2V_{o} Fs}} \hfill \\ L_{4} \ge \frac{{V_{g} DR_{L} }}{{V_{o} (1 - D)Fs}} \hfill \\ \end{gathered} \right\}$$

60 $$C_{2} = \frac{{V_{out} D}}{{\Delta VR_{L} Fs(1 - D)}} = C_{3}$$

61 $$C_{4} = \frac{VoD}{{\Delta Vc_{4} .FsRL}}$$

62 $$C_{5} = \frac{{V_{o} D}}{{\Delta V_{o} FsRL}}$$

Table 1 indicates that the utilization of a high switching frequency can decrease the size of inductors and capacitors in the proposed converter, leading to a lightweight and compact prototype with reduced costs. The specifications for the inductors include the use of flat wire with minimal internal resistance, effectively lowering losses in the proposed converter. Moreover, the power MOSFETs employed feature extremely low on-state resistance, thereby increasing voltage transfer gain and decreasing conduction power losses. In order to.

**Table 1:** Design of prototype components for proposed converter.

$SiC\; \, MOSFET$	$650\;v \, ,Ron = 57\;\text{m}\Omega$ (IMZA65R057M1H)
$SiC \, \;Schottcky$	${ 1200}\;{\text{V 40}}\;{\text{A,Vf = 1}}{.1}$ IDWD40G120C5
$$\begin{gathered} L1, \hfill \\ L2 \, \hfill \\ \, L3 \hfill \\ and\; \, L4 \hfill \\ \end{gathered}$$	$$\begin{gathered} 100\;\upmu {\text{H}},2.9\;{\text{m}}\Omega , \hfill \\ 2.2\;\upmu {\text{H}},1.3\;{\text{m}}\Omega \hfill \\ { 100}\;\upmu {\text{H}},2.9\;{\text{m}}\Omega \hfill \\ and \, 15\;\upmu {\text{H}},1.9\;{\text{m}}\Omega \hfill \\ \end{gathered}$$ (flat wire with very small size)
$$\begin{gathered} C1, \hfill \\ C2{ = }C3 \hfill \\ C4,andC5 \hfill \\ \end{gathered}$$	$$\begin{gathered} 2\;\upmu {\text{F }}100\;{\text{V}}, \hfill \\ 200\;\upmu {\text{F }}100\;{\text{V}} \hfill \\ 10\;\upmu {\text{F 500}}\;{\text{V}} \hfill \\ and,100\;\upmu {\text{F 500}}\;{\text{V}} \hfill \\ \end{gathered}$$
$V_{g}$	$20 - 40\;{\text{V}}$
$Vo$	$200 - {400}\;{\text{V}}$
$Power$	$200\;{\text{W}},400\;{\text{W}}$
$RL$	$200\;\Omega { ,400}\;\Omega$
Duty Cycle	$$\begin{gathered} {0}{\text{.27}}\;{\text{at }}\;{200}\;{\text{w}}\;{\text{ at}}\;{ 40}\;{\text{V }} \hfill \\ 0.33 \, \;at\;{ 4}00\;{\text{w}}\;{\text{ at }}\;{40}\;{\text{V}} \hfill \\ \end{gathered}$$
Frequency	$150\;{\text{kHZ}}$
Ic drive	1EDI60N12AF
Inductor size (L1,L3 and L4) L2 size	(L/2.5 cm*W/2.25 cm*H/1.78 cm) (L/1.5 cm*W/1.2*H/1.5 cm)
Input current ripple percentage	(10–25%)

## Comparison of the proposed converter with other high boosting converters

**Figure 5:**
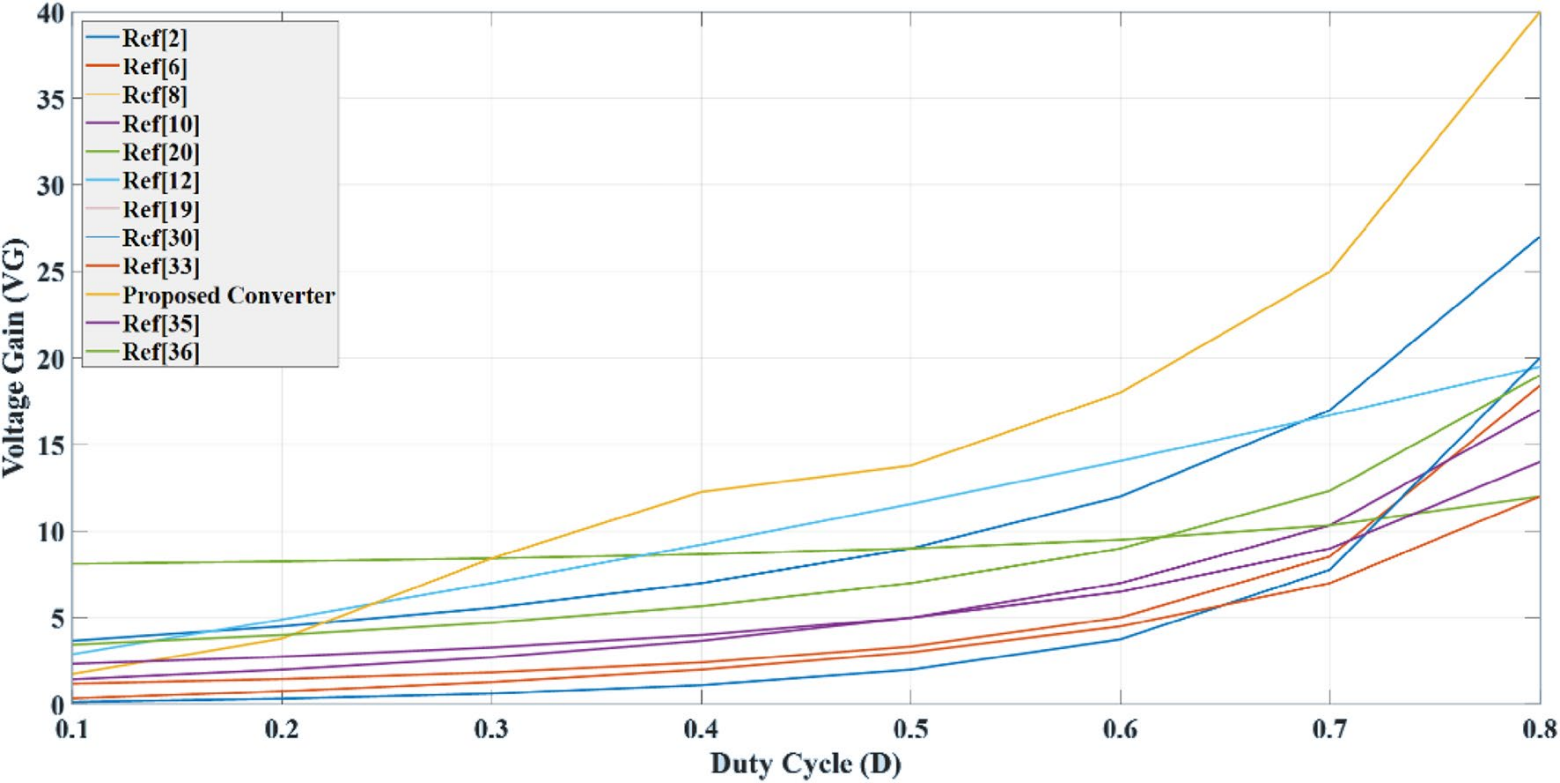
Comparison of voltage transfer gain of converters.

**Figure 6:**
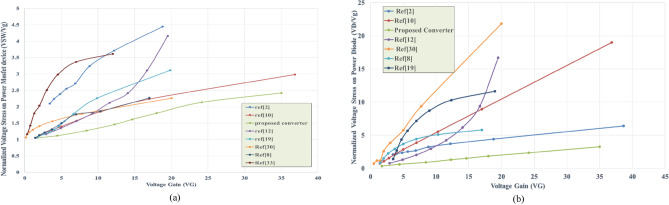
(**a**) Voltage stress across power MOSFET versus voltage gain, (**b**) voltage stress across power diode versus voltage gain.

To validate the superior performance of the proposed converter in achieving a high voltage gain and low voltage stress across power devices, a comparison is conducted in this section between the proposed converter and previous DC–DC converters. The previous DC–DC converters were simulated using Matlab Simulink under the same conditions. Figure 5 shows that a higher voltage transfer gain is exhibited by the proposed converter compared to the previous converters. Additionally, the high gain of the proposed converter at a low duty cycle indicates several benefits, including higher efficiency, lower switching losses, low voltage stress across power devices, lower conduction losses with rms value current, and fewer capacitors and inductors required to achieve high voltage gain. The voltage gain of the proposed converter, approximately 13.5, can be calculated using Eq. (42) when the converter operates in DCM2.When looking at Fig. 6a, it can be seen that the voltage across the MOSFET device in the proposed converter is lower than that in the previous converters when compared to the gain ratio. In Fig. 6b, it can be observed that the voltage across the diode to the voltage source in the proposed DC–DC converter is lower than that in the previously used boosting converters.

Table 2 presents a comparison between the proposed DC–DC converter and previous converters. The comparison focuses on the number of inductors, capacitors, diodes, switching frequency, input current, duty cycle percentage, and MOSFETs utilized in both the proposed and previous boosting converters. The results indicate that the previous boosting DC–DC converters are operated at a lower switching frequency than the proposed converter, resulting in higher inductor and capacitor values, as well as higher parasitic resistance. Additionally, the MOSFET's internal resistance at low switching frequency is high, leading to high conduction and switching losses. Moreover, the values of inductors and capacitors used in previous converters are larger, resulting in a larger, more costly, and heavier system. The previous boosting DC–DC converters can boost low voltage with a high voltage transfer ratio, but at an extremely high duty ratio. In contrast, the proposed converter can boost low voltage sources to a variable output voltage between 200 and 400 V at a lower duty ratio than previous boosting converters. The input current in the proposed converter is non-pulsating at both low and high duty cycles, whereas the converters in References^2, 6, 10, 12^ exhibit pulsating currents at low duty cycles. Finally, the proposed converter has a higher voltage gain compared to previous boosting DC–DC converters, making it more suitable for renewable energy systems that require variable and fixed high output voltage with a wider range in the duty ratio.

**Table 2:** A comparative analysis of the proposed converters and previous high-boosting converters.

Items	Proposed converter	Reference^2^	Reference^6^	Reference^8^	Reference^10^	Reference^20^	Reference^12^	Reference^19^	Reference^30^	Reference^33^	Reference^35^	Reference^36^
F_S_ (KHZ)	150	100	30	30	50	100	1	24	24	30	50	50
Vg	20–40v	10v	60v	29v	40v	40v	24v	20v	20v	25v	25v	48v
Vo	200v 400v	120v	300v	325v	260v	364v	221v	300v	172.2v	110v	200v	408v
L	4	3	5	4	3	4	4	2	3	4	2	2
C	5	6	5	6	3	7	2	4	3	6	5	3
Diodes D	4	6	0	4	2	3	8	4	3	3	4	3
Switches SW	2	1	7	2	2	2	2	1	1	1	1	2
Duty cycle	33%	73.5%	70%	73%	72%	71%	42%	77%	70%	60%	72%	60%
Power	400w	50 w	200w	220w	200w	265w	200w	250w	100w	110w	200w	250w
Input current	No pulsating	Pulsating	Pulsating	No pulsating	Pulsating	No pulsating	Pulsating	No pulsating	No pulsating	Pulsating	No pulsating	Pulsating at low D
Efficiency %	96.3	88	93	95.5	96	94.5	92	91.5	91.4	92	93	91
VG at D = 0.75	24	21	12.4	13	13	11	17.2	13.5	12	9	11	15
$VG = \frac{Vo}{{Vg}}$	$\frac{2D}{{(1 - D)^{2} }}$	$\frac{{3\left( {1 + D} \right)}}{{\left( {1 - D} \right)}} \,$	$\frac{{(1 - \frac{1}{3}D)}}{{(1 - D)^{2} }}$	$\frac{{\left( {1 + 3D} \right)}}{{\left( {1 - D} \right)}}$	$\frac{{\left( {1 + 3D} \right)}}{{\left( {1 - D} \right)}}$	$\frac{{\left( {8 - 7D} \right)}}{{\left( {1 - D} \right)}}$	$\frac{(1 + 18.25D)}{{(1 - 0.25D)}}$	$\frac{(3 + D)}{{2(1 - D)}}$	$\frac{\left( D \right)}{{\left( {1 - D} \right)^{2} }}$	$\frac{{\left( {3D} \right)}}{{\left( {1 - D} \right)^{{}} }}$	$\frac{{\left( {2 + D} \right)}}{{\left( {1 - D} \right)}}$	$\frac{(3 + D)}{{\left( {1 - D} \right)}}$

## Control strategy of the proposed converter

**Figure 7:**
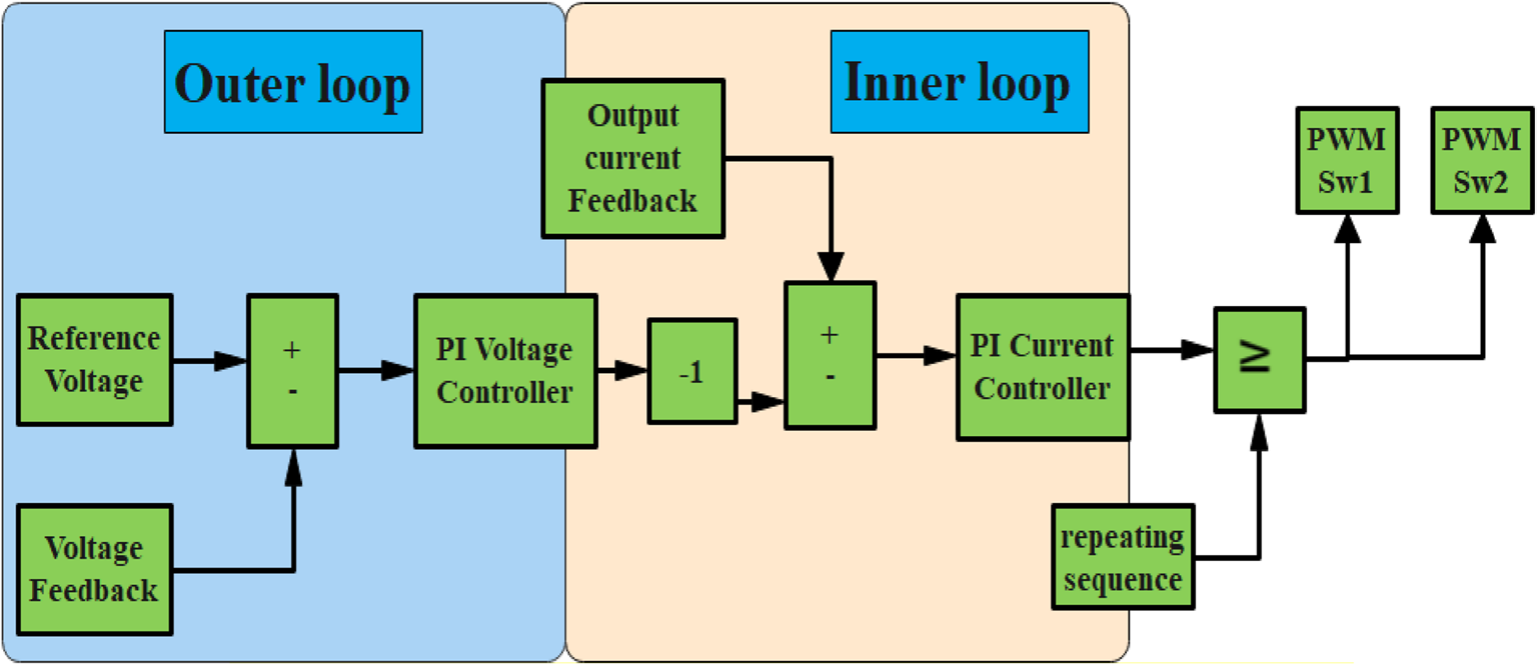
Voltage and current controller of proposed converter.

**Figure 8:**
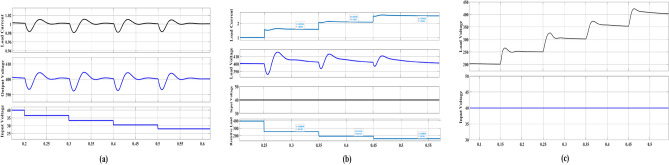
(**a**) Output of proposed converter at variable input voltage, (**b**) variable load current at fixed output and input voltage of the proposed converter, (**c**) variable load voltage 200–400 V at fixed input voltage.

The proposed converter controller, as depicted in Fig. 7, employs double PI controllers. The first controller serves as the inner loop controller and is designed to regulate the load current, while the second controller functions as the outer loop controller and is responsible for regulating the output voltage. The PI voltage controller takes the error between the reference voltage and the output voltage as its input, and its output is the reference current, which is restricted to prevent excessive current draw from the converter. The error between the reference current and the output current is then fed into the PI current controller. The controller design process involves utilizing an equation that describes a PI controller with proportional and integral action control parameters KP and Ki. In Fig. 8(a), the proposed converter can maintain a constant output voltage of 400 V, regardless of input voltage changes from 40 to 30 V. This exemplary performance highlights the stability of the proposed converter and its capacity to deliver a consistent output voltage despite varying input voltage levels. In Fig. 8(b), the proposed converter can validate a constant output voltage at a fixed input voltage with variable load current. In Fig. 8(c), it can be observed that the proposed converter can verify variable output voltage ranging from 200 to 400 V at a fixed input voltage. This makes the proposed converter more suitable for a wide range of applications.

The dual PI controller of the proposed converter demonstrates enhanced robustness and reliability, facilitating a quicker attainment of steady state. Furthermore, the proposed converter, equipped with current and voltage controllers, can achieve a wide range of duty ratios to supply high load current.

## Results and discussions of proposed converter: simulation and experiment

**Figure 9:**
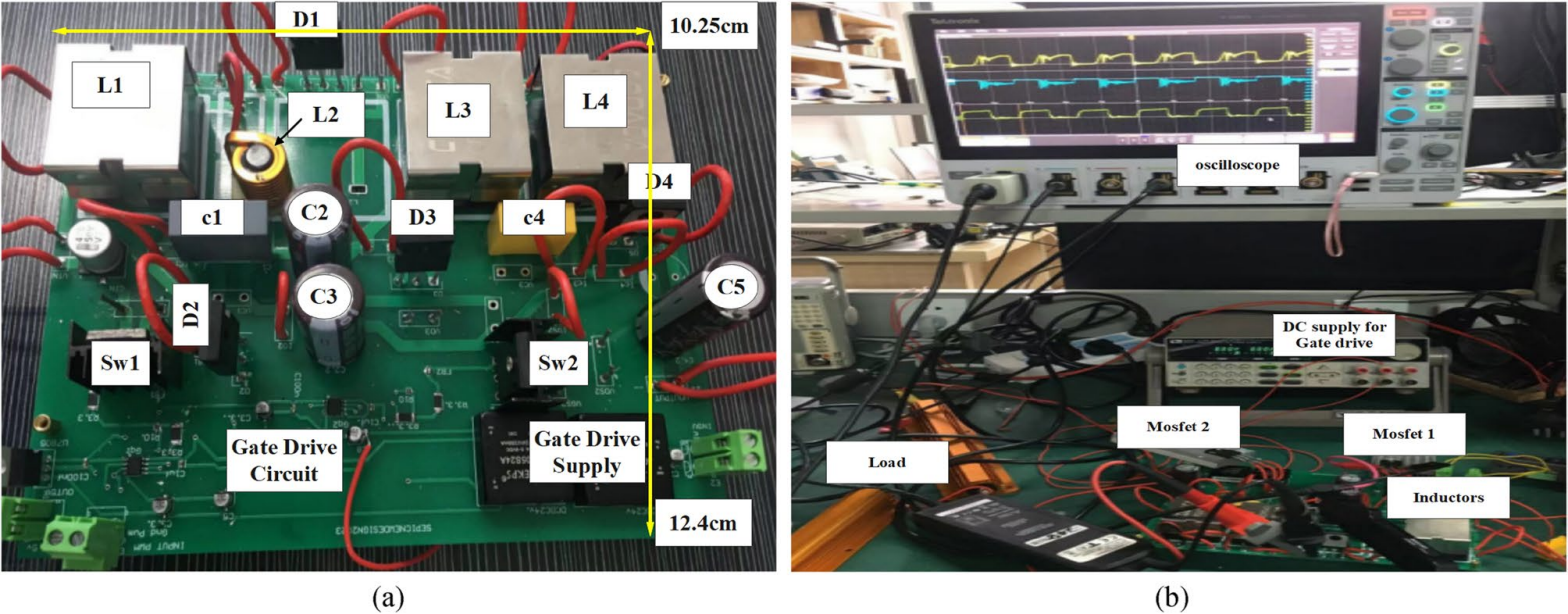
(**a**) PCB design of the prototype proposed converter, (**b**) experimental prototype test of the proposed converter.

In this section, a prototype of the 400 W converter design was constructed to confirm the accuracy of the simulation and experimental outcomes, as depicted in Fig. 9a. The converter was subjected to experimental evaluation in the laboratory, as shown in Fig. 9b. Furthermore, the laboratory results were verified using MATLAB software under various conditions. It should be noted that non-ideal inductors and capacitors were used, and all parasitic resistances were accounted for in the proposed DC–DC converter.

**Figure 10:**
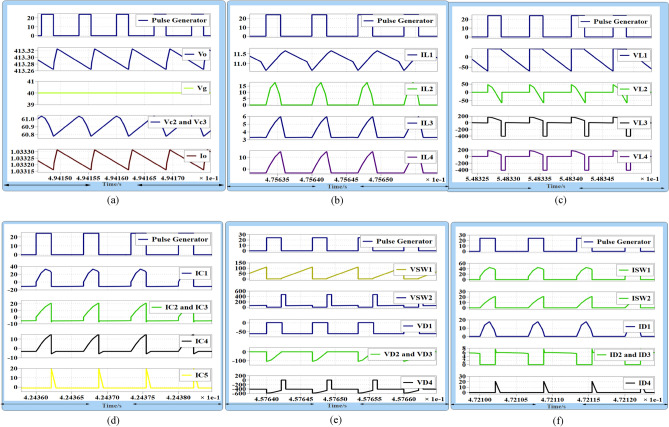
Proposed converter operation at DCMC1 (**a**) gate source voltage at D = 0.33, Vo = 413 V, Vc_2_ = 60 V, Vg = 40 V and load current Io = 1 A, (**b**) iL_1_, iL_2_, iL_3_ and iL_4_, in DCMC1, (**c**) VL_1_, VL_2_, VL_3_ and VL_4_, (**d**) capacitors Current Ic_1_, Ic_2_, Ic_3,_ Ic_4_ and Ic_5_, (**e**) voltage stress, Vsw_1_, Vsw_2_ and VD_1_, VD_2_, VD_3_ and VD_4_, (**f**) Isw_1_, Isw_2_ I_D1_, I_D2_, I_D3_ and I_D4._

In Fig. 10a depicts a PWM generator producing a 33% duty ratio with an output voltage of 413 V. The voltage source is 40 V, and the voltage across capacitors C_2_ is 60 V at a load current of 1 A. Figure 10b displays the current flowing through inductors L_1_, L_2_, L_3_, and L_4_. It is evident that iL_1_ experiences no pulsation at the low duty cycle of 33% with very low ripple input current. Current L_2_ discharges to C_2_ and C_3_ during period (m1), while the currents through L_3_ and L_4_ have the same shape but in reverse directions. Figure 10c shows the voltage across inductors. It is noticeable that the voltage across L_2_ is Vc_2_ during the off state of period (m1), and it is zero from (D + m1 < t < Ts). Figure 10d shows the current through capacitors, while Fig. 10e displays the voltage across power MOSFETs and diodes of the proposed converter. The voltage stress across S_W1_ is significantly reduced, and the average voltage is equal to the input voltage. The voltage across Sw_2_ is equal to the output voltage during the very short period (D < t < D1) and Vc_3_ voltage during the very long time (D1 < t < Ts). Moreover, the voltage across D_1_, D_2_, and D_3_ is equal to Vc_3_. Figure 10f illustrates the current through MOSFETs and power diodes. It can be observed that IS_W1_ equals iL_1_ and iL_2_, while the current through IS_W2_ is equal to capacitor current C_2_. Furthermore, the current through D_2_ and D_3_ equals L_1_ and L_2_ current during the off state.

**Figure 11:**
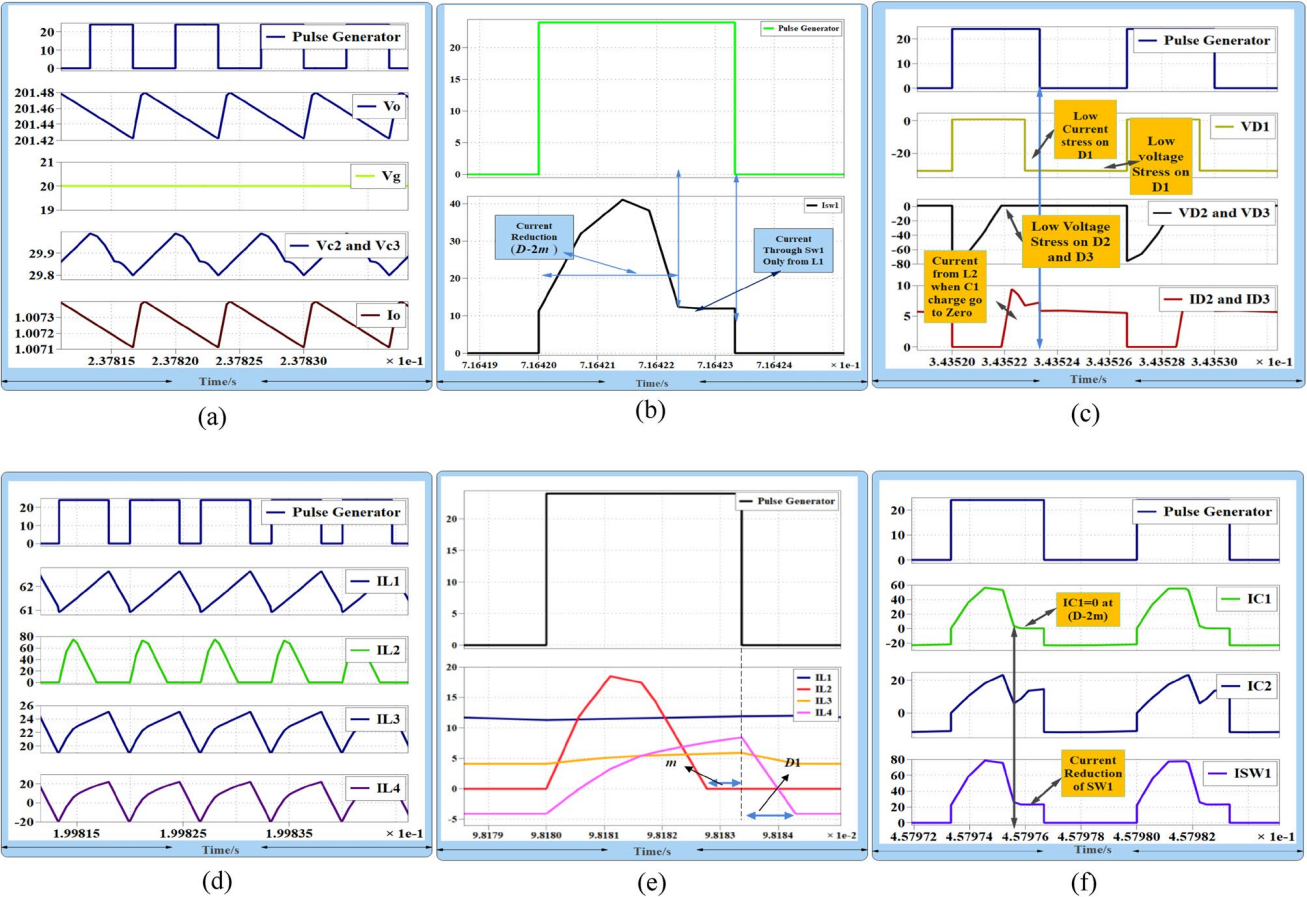
(**a**) Gate source voltage at D = 0.5, Vo = 200 V, Vc_2_ = 29 V, and Vg = 20 V, (**b**) Isw_1_, at zero charge of C1 and show the current reduction of Sw1 after (D-2m), (**c**) V_SW1_,V_SW2_ and V_D1_,V_D2_,V_D3_ and V_D4_, (**d**) current through inductors iL_1_,iL_2_,iL_3_ and iL_4_, at CCM, (**e**) current through inductors at DCMC2, (**f**) current through capacitors IC_1_ and IC_2_ and current through S_w1_.

During this experiment, the voltage transfer gain equals 10 at a duty cycle of 0.33. The proposed converter exhibits zero pulsating input current at very low duty ratios. Additionally, the voltage across power MOSFETs and diodes is significantly reduced, indicating very small values. Hence, the proposed DC–DC converter is more suitable for renewable energy sources (RES) applications. After incorporating all parasitic components, the simulations show that the proposed converter operates in DCMC2. At an input voltage of 20 V, Fig. 11a demonstrates that the converter produces an output voltage of 200 V, and the voltage across capacitor Vc_3_ is approximately 29 V at a load current of 1 A. Figure 11b displays the current through power switch S_W1_, revealing that the stress current through S_W1_ is reduced because charge in C_1_ becomes zero at (D-2m) and L_2_'s charge makes D_2_ and D_3_ turn on to charge L_3_ and L_4_. Figure 11c shows the voltage across D_2_ and D_3_. It can be seen that the voltage across D_2_ and D_3_ is totally reduced when L_2_ and C_1_ work in resonant mode. So, both of them opearte in ZVS during this time (D-2m) as shown in Fig. 13c. Meanwhile, L_2_ will pass current through them at (D-2m) < t < Ts and will work as ring to charge L_3_ and L_4_ through S_w2_. Figure 11d shows inductor current when the converter operates in CCM, and Fig. 11e shows inductor current in DCMC2.

**Figure 12:**
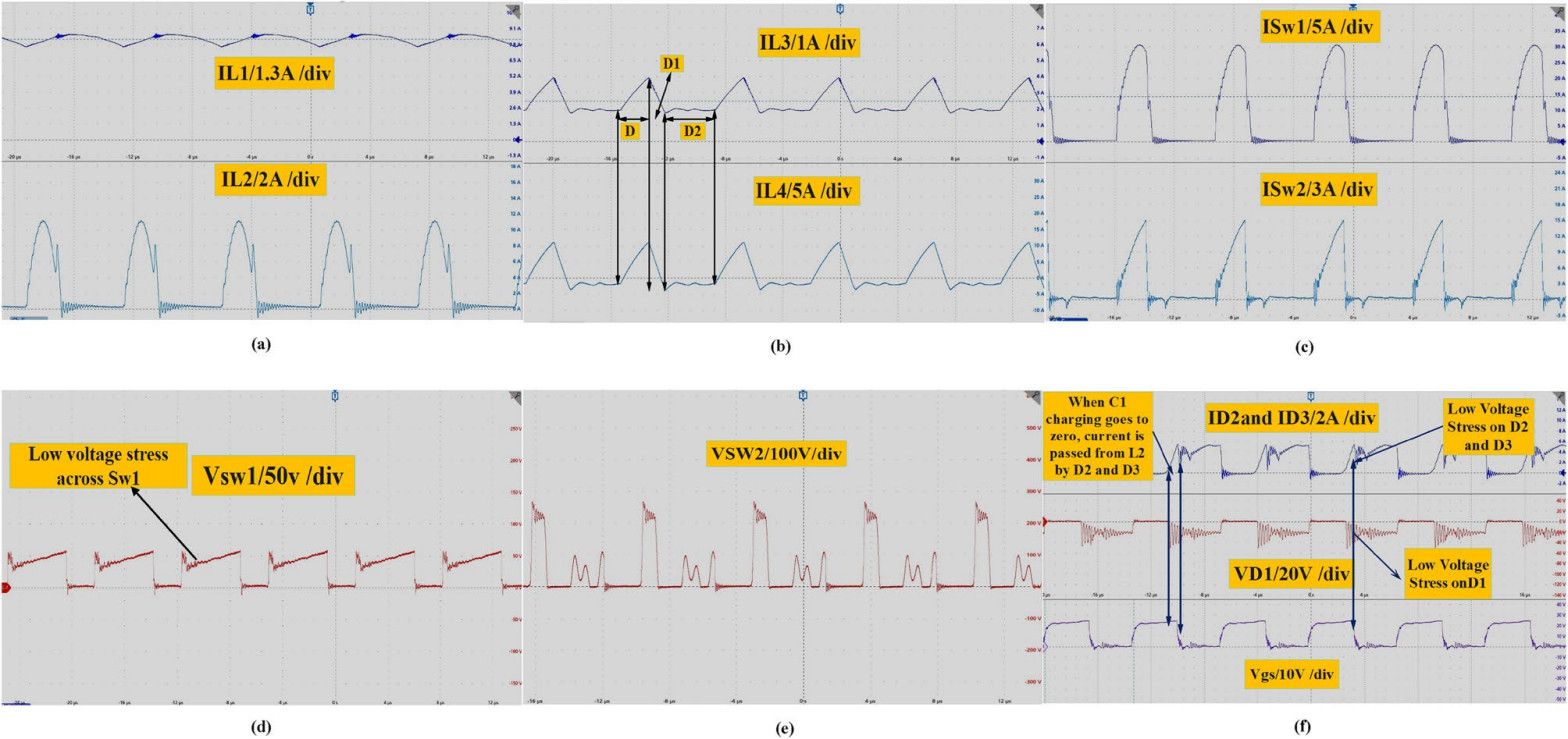
(**a**) Inductors currents iL_1_, iL_2_ at DCMC1, (**b**) inductors currents iL_3_ and iL_4_, at DCMC1, (**c**) power MOSFETs current Is_w1_, Is_w2_, (**d, e**) voltage across power MOSFET Vs_w1_ and Vs_w2_, (**f**) current through D_2_,D_3_ and Voltage across D_1_.

**Figure 13:**
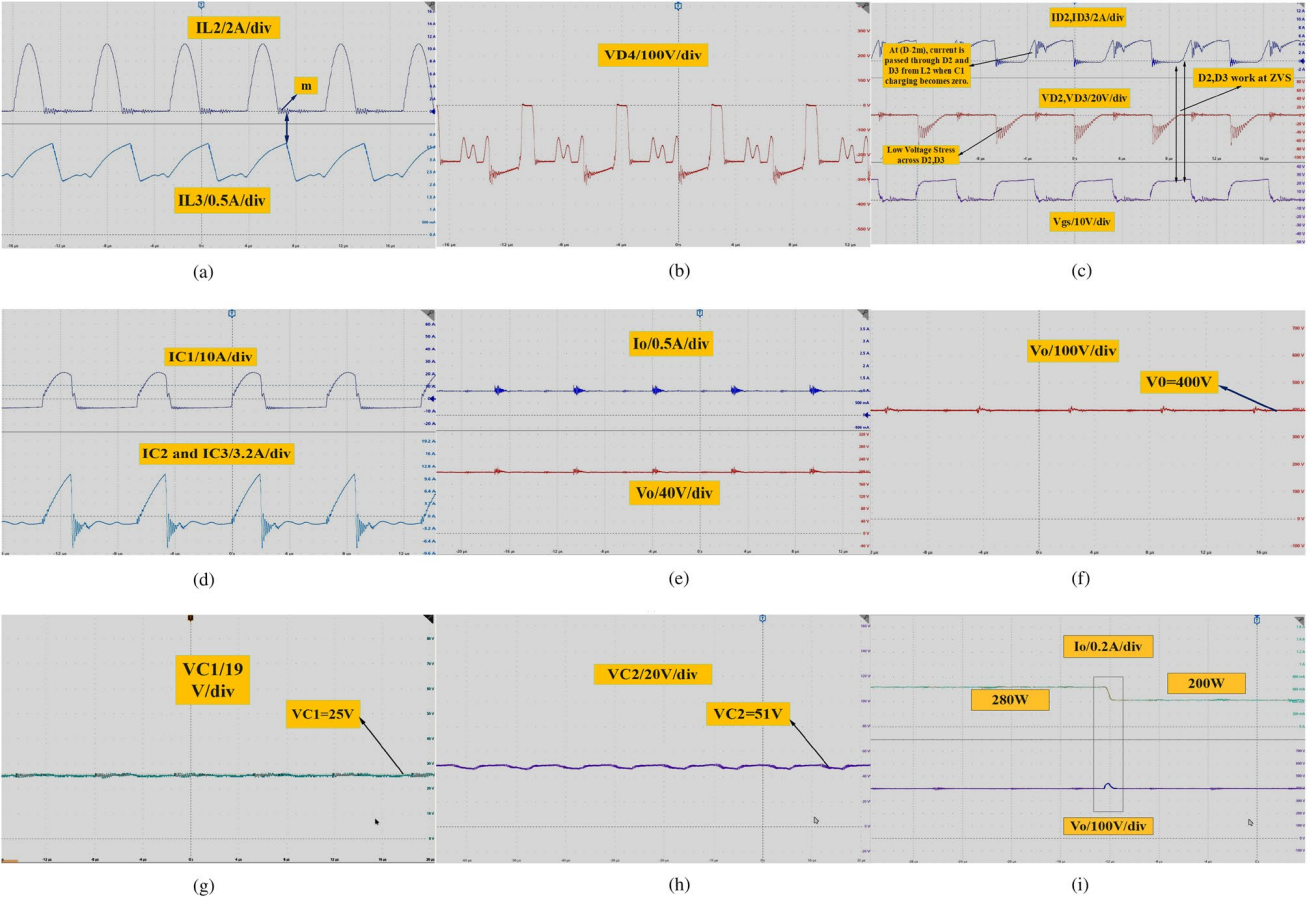
(**a**) Inductors currents iL_1_,iL_2_ at DCMC2, (**b**) VD_4_, (**c**) current through D_2_, D_3_ and voltage across D_2_ and D_3_, (**d**) current through capacitors C_1_ and C_2_,C_3_, (**e**) Vo = 200 V at load current 1 A, (**f**) load voltage V_O_ = 400 V. (**g**) Vc1, (**h**) Vc2 and Vc3, (**i**) dynamic response of the proposed converter at load change from 280 to 200 w.

Figure 11f shows that the current through capacitors Ic_1_ and Ic_2_, as well as the current through S_w1_, reduce when the charge in capacitor C_1_ becomes zero at (D-2m). In terms of experimental test results, Fig. 12a illustrates the current through inductors L_1_ and L_2_ when the converter operates in DCMC1. Figure 12b illustrates the current through inductors L_2_ and L_3_ when the converter operates in DCMC1. Figure 12c shows the MOSFETs current IS_W1_ and IS_W2_. Figure 12d,e show the voltage across switches Vs_w1_ and Vs_w2_. In Fig. 12f, the current through D_2_ and D_3_ can be observed when L_2_ discharges current through them due to the charge in capacitor C_1_ becoming zero at (D-2 m). It shows that D_1_ operates with low current stress, and during this time, inductor L_2_ works as an open circuit. The voltage across D_1_ is equal to Vc_3_. Figure 13a displays the inductor current when the converter operates in DCMC2. In Fig. 13b, the voltage across D_4_ is shown.

Figure 13c demonstrates the voltage across D_2_ and D_3_, revealing that they experience ZVS during the on state from (D-2m) when charge in C_1_ becomes zero during (D-2m). During this time, L_2_ discharges current to charge L_3_ and L_4_ through S_w2_, reducing the voltage across the power MOSFET and diodes. This method enhances the efficiency and performance of the converter. Furthermore, the open circuit state of inductor L_2_ minimizes losses from the converter's passive components. Figure 13d shows the currents through capacitors IC_1_ and IC_2_. Figure 13e displays the output voltage of the proposed converter, Vo = 200 V, at a load current of 1 A, with D = 50%. Figure 13f shows the load voltage Vo = 400 V at D = 33%. In Fig. 13g, the voltage across C_1_, equal to 25 V, is depicted, while Fig. 13h shows the voltage across C_2_ at a low duty cycle, which is equal to 54 V. The dynamic response of the proposed converter at load changing from 280 to 200 w is shown in Fig. 13i.

As a result, when the input voltage reaches its minimum value, the proposed converter can operate with high efficiency. It utilizes D_1_ power diode, which operates with low current stress and low voltage stress, and diodes D_2_ and D_3_, which work at low voltage stress during the on state, thus reducing the number of passive components. Additionally, this design enhances the efficiency and reliability of the proposed converter for renewable energy systems that require high output fixed and variable DC voltages. Furthermore, the voltage stress across S_w1_ and S_w2_ is significantly reduced when the proposed converter operates in DCMC2 at 400 W. Moreover, the current and voltage stress of all power diodes are significantly reduced, resulting in a significant reduction in total power losses. Additionally, the current of S_w1_ is significantly reduced when a single cell of switched inductor capacitors is added and operates in the resonant mode.

## Analyzing power loss and efficiency of the proposed converter

**Figure 14:**
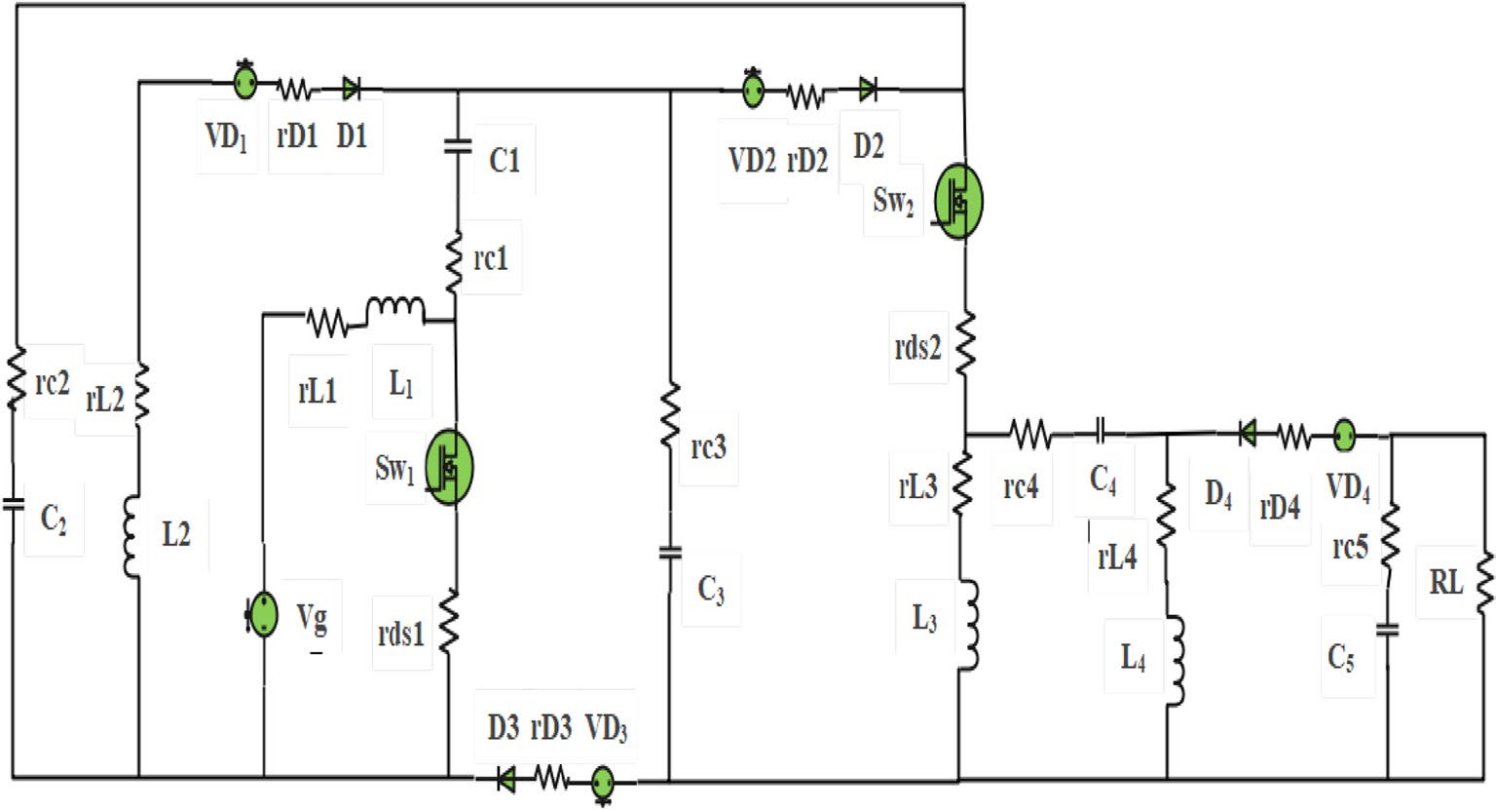
The proposed converter with internal resistance in passive and active components.

The proposed converter comprises five capacitors, four inductors, two power switches, and four diodes. However, it should be noted that these components are not ideal, meaning that each component has its internal resistance that affects its performance. For instance, the internal resistance of an inductor increases as the value of the inductor increases. The internal resistance of each inductor is denoted as rl1, rl2, rl3, and rl4, while the equivalent series resistances of capacitors are rc1, rc2, rc3, rc4, and rc5. Additionally, the power diode has two power losses: one due to its internal resistance rd and the other due to its forward voltage Vf. Other power losses occur due to conduction and switching losses of the power MOSFET devices. Therefore, all of these losses should be taken into account when considering the proposed converter. The internal resistances of all the inductors, capacitors, power diode, and MOSFETs can be seen in Fig. 14.

63 $$Irms = \sqrt {\frac{1}{Ts}\int\limits_{0}^{Ts} {(I)^{2} dt} }$$

64 $$Isw_{1rms} = \sqrt {\frac{1}{Ts}\int\limits_{0}^{(D - 2m)Ts} {(iL_{1} + iL_{2} )^{2} dt + \int\limits_{(D - 2m)T}^{DTs} {(iL_{1} )^{2} dt + } } }$$

65 $$Isw_{2rms} = \sqrt {\frac{1}{Ts}\int\limits_{0}^{(D-2m)} {(Ic2)^{2} + \int\limits_{(D - 2m)}^{(D - m)} {(Ic2+Ic3)^{2} } dt} }$$

66 $$\scriptsize ID_{1rms} = \sqrt {\frac{1}{Ts}\int\limits_{0}^{(D - m)Ts} {(iL2)^{2} dt} } = iL2rms$$

67 $$ID_{2rms} = ID_{3rms} = \sqrt {\frac{1}{Ts}\int\limits_{(D - 2m)}^{DTs} {(iL_{2} )^{2} dt + \int\limits_{DTs}^{Ts} {(iL_{1} )^{2} dt} } }$$

68 $$ID_{4rms} = \sqrt {\frac{1}{Ts}\int\limits_{D}^{Ts} {(iL_{3} + iL_{4} )^{2} dt} }$$

69 $$Ic_{1rms} = \sqrt {\frac{1}{Ts}\left[ {\int\limits_{0}^{(D - 2m)Ts} {(iL2)^{2} dt + \int\limits_{D}^{Ts} {(iL1)^{2} dt} } } \right]}$$

70 $$Ic_{2rms} = Ic_{3rms} = \sqrt {\frac{1}{Ts}\left[ {\int\limits_{0}^{DTs} {(iL3 + iL4)^{2} dt + \int\limits_{D}^{Ts} {(iL1)^{2} dt} } } \right]}$$

71 $$Ic_{4rms} = \sqrt {\frac{1}{Ts}\left[ {\int\limits_{0}^{DTs} {(iL4)^{2} dt + \int\limits_{D}^{Ts} {(iL3)^{2} dt} } } \right]}$$

72 $$Ic_{5rms} = \sqrt {\frac{1}{Ts}\left[ {\int\limits_{0}^{DTs} {(I_{o} )^{2} dt + \int\limits_{D}^{Ts} {(iL3 + iL4)^{2} dt} } } \right]}$$

To determine the total power losses of the proposed converter, it is necessary to calculate the RMS current for the inductors, capacitors, power MOSFET, and diodes in both on and off states. Equation (63) represents the general equation for RMS current. Equations (64) and (65) can be utilized to obtain the RMS current through S_W1_ and S_W2_ during the on state. During the on state, the RMS current through D1, which is equal to the RMS current through inductor L_2_ during the period (0 < t < D-m), can be calculated using Eq. (66). Equation (67) provide the RMS current for D_2_ and D_3_ and, Eq. (68) provide the RMS current for D4, and Eqs. (69), (70), (71),and (72) can be employed to determine the RMS currents for capacitors C_1_, C_2_, C_3_, C_4_ and C_5_, respectively.

73 $$Isw_{1rms} = \frac{{2Io\sqrt {4D - 6m} }}{{(1 - D)^{2} }}$$

74 $$Isw_{2rms} = \frac{{2DIo\sqrt {D + m} }}{{(1 - D)^{2} }}$$

75 $$ID_{1rms} = \frac{{2IoD\sqrt {D - m} }}{{(1 - D)^{2} }} = iL_{2rms}$$

76 $$ID_{2rms} = ID_{3rms} = \frac{IoD}{{(1 - D)^{2} }}\sqrt {2m + 1 - D}$$

77 $$ID_{4rms} = \frac{Io(1 + D)}{{\sqrt {(1 - D)} }}$$

78 $$iL_{1} rms = \frac{2IoD}{{(1 - D)^{2} }}$$

79 $$iL_{3rms} = \frac{2DIo}{{(1 - D)}}$$

80 $$iL_{4rms} = \frac{DIo}{{(1 - D)}}$$

81 $$Ic_{1}^{{}} rms = \frac{{IoD\sqrt {1 + 2m_{{}} } }}{{(1 - D)^{2} }}$$

82 $$Ic_{2rms} = Ic_{3rms} = \frac{IoD}{{(1 - D)^{2} }}\sqrt {2m + 1 - D}$$

83 $$Ic_{4rms} = Io\sqrt D \sqrt {\frac{1 + 3D}{{1 - D}}}$$

84 $$Ic_{5rms} = \frac{Io(1 + D)}{{\sqrt {(1 - D)} }}$$

Equations (73) and (74) describe the RMS current flowing through the MOSFETs. To calculate the RMS currents flowing through the power diodes, you can use Eqs. (75), (76), and (77). Equations (78), (79), and (80) provide the RMS current flowing through inductors L_1_, L_3_, and L_4_ respectively. Finally, we can determine the RMS current through capacitors C_1_, C_2_, C_3_, C_4_, and C_5_ using Eqs. (81), (82), (83), and (84), respectively.

### Conduction and switching losses calculation of power MOSFETs

To determine the conduction losses of the power MOSFET in the proposed converter, (RMS) current is multiplied by the value of the on state resistance of the MOSFET.

85 $$Pcd1 = \frac{4Po(4D - 6m)}{{R_{L} (1 - D)^{4} }}rds1$$

86 $$Pcd2 = \frac{{4P_{o} D^{2} (D + m)}}{{RL(1 - D)^{4} }}rds2$$

87 $$PSW = \frac{1}{2}Vsw^{2} FsCo$$

88 $$P_{SWL1} = \frac{1}{2}V_{g}^{2} FsCo$$

89 $$P_{SWL2} = \frac{{V_{o}^{2} (1 - D)^{2} }}{{8D^{2} }}FsCo$$

90 $$T_{PLCS1,2} = \frac{4Po(4D - 6m)}{{R_{L} (1 - D)^{4} }}rds1 + \frac{{4P_{o} D^{2} (D + m)}}{{RL(1 - D)^{4} }}rds2 + \frac{1}{2}V_{g}^{2} FsCo + \frac{{V_{o}^{2} (1 - D)^{2} }}{{8D^{2} }}FsCo$$

The power conduction losses of MOSFETs Sw_1_ and Sw_2_, Pcd1 and Pcd2, can be obtained from Eqs. (85) and (86). Equation (82) provides a means to calculate the power switching losses, P_SWL1_ and P_SWL2_, of MOSFETs Sw_1_ and Sw_2_. These losses can be obtained using the output capacitor of the MOSFET, Co, and the switching frequency, Fs. The total power losses of MOSFETs P_SWL1_ and P_SWL2_ can be obtained by adding Eqs. (85), (86), (88), and (89) as a result, resulting in Eq. (90).

### Power losses calculation in all diodes

In order to accurately calculate the power losses incurred by the four diodes in the converter, it is important to take both types of losses into consideration. To determine the power losses due to Vf, one can use Eq. (91) to calculate the average current flowing through diodes, and then multiply this result by Vf and the power losses (Pvf) caused by the forward voltage. On the other hand, Eq. (92) can be used to calculate the diode power losses due to rd, while Eq. (93) is used to determine the power losses due to Vf. Finally, to obtain the total power losses (T_PDL1_, T_PDL2_, T_PDL3_, T_PDL4_) across all four diodes, Eq. (94) can be used to add up all losses in the power diode.

91 $$\left. \begin{gathered} I_{D1ave} = \frac{{2IoD(D - m_{{}} )}}{{(1 - D)^{2} }} \hfill \\ I_{D2ave} = \frac{{2I_{o} ( - D + m + 1)}}{{(1 - D)^{2} }} \hfill \\ I_{D3ave} = \frac{{2I_{o} ( - D + m + 1)}}{{(1 - D)^{2} }} \hfill \\ I_{D4ave} = I_{o} (1 + D) \hfill \\ \end{gathered} \right\}$$

92 $$\left. \begin{gathered} P_{Dr} = IDrmsrd \hfill \\ P_{Dr1} = \frac{{4P_{o} D^{2} (D - m_{{}} )}}{{RL(1 - D)^{4} }}rd1 \hfill \\ P_{Dr2} = \frac{PoD(2m + 1 - D)}{{R_{L} (1 - D)^{4} }}rd2 \hfill \\ P_{Dr3} = \frac{PoD(2m + 1 - D)}{{R_{L} (1 - D)^{4} }}rd3 \hfill \\ P_{Dr4} = \frac{{P_{o} (1 + D)^{2} }}{{R_{L} (1 - D)}}rd4 \hfill \\ \end{gathered} \right\}$$

93 $$\left. \begin{gathered} P_{Vf} = I_{Dav} Vf \hfill \\ P_{Vf1} = Vf_{1} \frac{{2P_{o} D(D - m_{{}} )}}{{RL(1 - D)^{2} }} \hfill \\ P_{Vf2} = Vf_{2} \frac{{2P_{o} ( - D + 1 + m)}}{{RL(1 - D)^{2} }} \hfill \\ P_{Vf3} = Vf_{3} \frac{{2p_{o} ( - D + 1 + m)}}{{RL(1 - D)^{2} }} \hfill \\ P_{Vf4} = Vf_{4} \frac{{P_{o} }}{RL}(1 + D) \hfill \\ \end{gathered} \right\}$$

94 $$T_{PDL} = P_{Dr1,2,3,4} + P_{Vf1,2,3,4}$$

### Total power losses in inductor and capacitors

95 $$\left. \begin{gathered} P_{L} = iLrms^{2} rl \hfill \\ P_{L1} = \frac{{4P_{o} D^{2} }}{RL(1 - D)4}rl1 \hfill \\ P_{L2} = \frac{{2P_{o} D^{2} (D - m)}}{{RL(1 - D)^{4} }}rl2 \hfill \\ P_{L3} = \frac{{4D^{2} P_{o} }}{{RL(1 - D)^{2} }}rl3 \hfill \\ P_{L4} = \frac{{D^{2} P_{o} }}{{RL(1 - D)^{2} }}rl4 \hfill \\ \end{gathered} \right\}$$

96 $$\left. \begin{gathered} P_{C} = Icrms^{2} rc1 \hfill \\ P_{C1} = \frac{{P_{o} D^{2} (1 + 2m)}}{{RL(1 - D)^{4} }}rc1 \hfill \\ P_{C2} = \frac{{PoD^{2} (2m + 1 - D)}}{{R_{L} (1 - D)^{4} }}rc2 \hfill \\ P_{C3} = \frac{{PoD^{2} (2m + 1 - D)}}{{R_{L} (1 - D)^{4} }}rc3 \hfill \\ P_{C4} = \frac{{P_{o} D(1 + 3D)}}{RL(1 - D)}rc4 \hfill \\ P_{C5} = \frac{{P_{o} (1 + D)^{2} }}{RL(1 - D)}rc5 \hfill \\ \end{gathered} \right\}$$

### Total power losses in proposed converter (T_PLPC_)

By utilizing Eqs. (95) and (96), it is possible to determine the power losses (PL) and (PC) in the inductors and capacitors, respectively. The losses associated with the proposed converter can be categorized into four types: MOSFET losses, diode losses, inductor losses, and capacitor losses. Equation (97) can be employed to calculate the total power loss (T_PLPC_) of the converter by adding the power losses of the power MOSFETs (T_PLCS1,2_), the total power losses of the diodes (T_PDL1,2,3,4_), and the losses in the inductors and capacitors (T_PL1,2,3,4_ and T_PC1,2,3,4,5_) respectively. The efficiency of the proposed converter can be determined by Eq. (98). Clearly, SiC MOSFETs with very low on-state resistance are the optimal choice for minimizing conduction losses. Additionally, utilizing inductors with low internal resistance values will further improve the performance and efficiency of the proposed converter.

97 $$T_{PLPC} \, = {\text{ T}}_{PLCS1,2} \, + {\text{ T}}_{PDL1,2,3,4} \, + {\text{ T}}_{PL1,2,3,4} + {\text{ T}}_{PC1,2,3,4} ,_{5}$$

98 $$\eta = \frac{{P_{o} }}{{P_{o} + T_{PLPC} }}100\%$$

**Figure 15:**
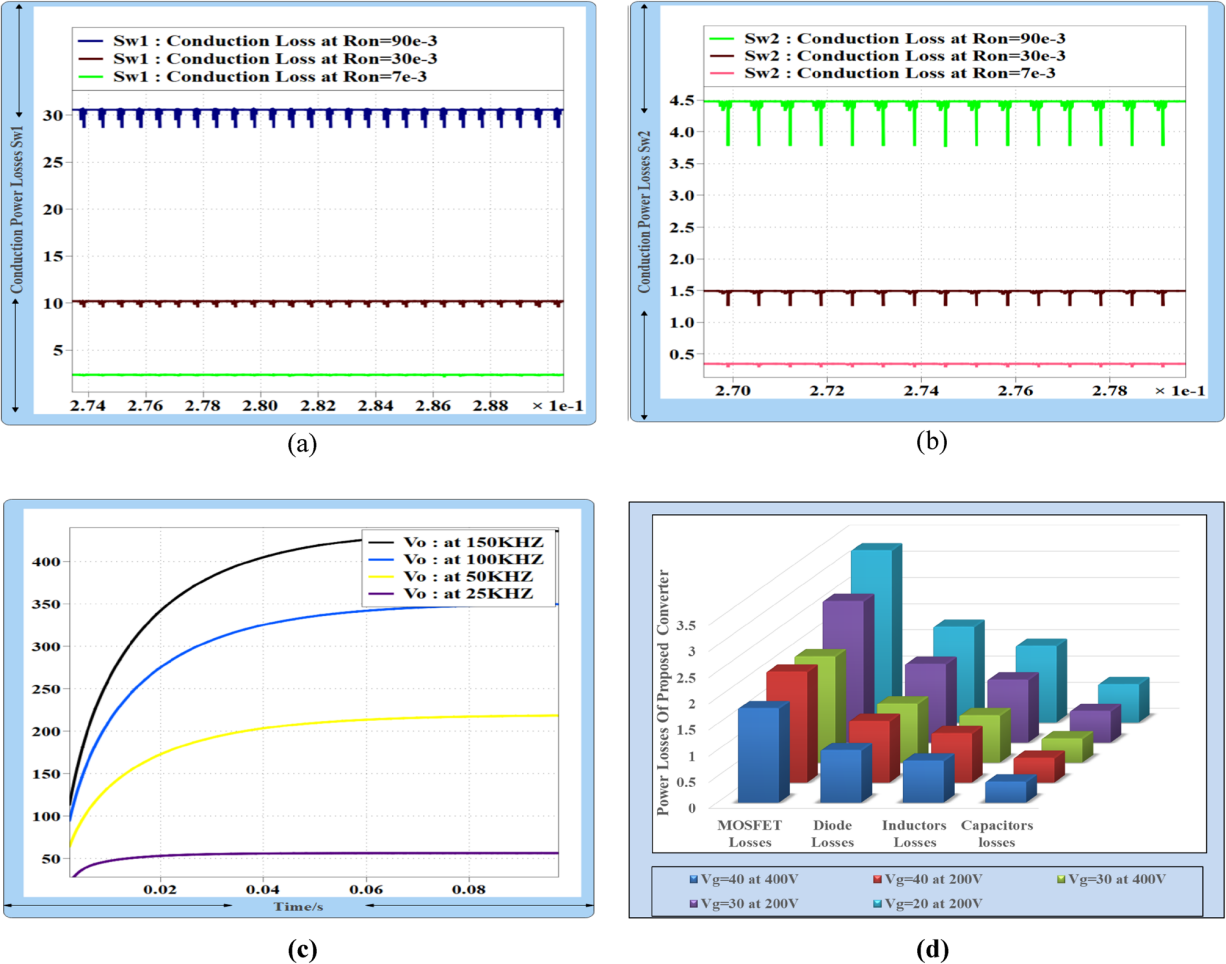
(**a, b**) Conduction power losses of MOSFET Sw1 and Sw2at variable input and output voltage, (**c**) output voltage of the proposed converter at different switching frequency at D = 33%, (**d**) total power losses in proposed converter.

Figure 15a,b illustrate the conduction power losses of MOSFETs across different input and output voltages. It showed that, the conduction power losses of both MOSFET with a small value of internal resistance are decreased.

Therefore, the proposed converter utilizing SiC MOSFET with Ron 7 m demonstrated minimal power conduction losses even with varying duty cycles, resulting in higher efficiency than previous DC–DC converters. The implementation of WBG MOSFETs is expected to further reduce both conduction and switching losses, leading to a substantial increase in the efficiency of the proposed converter.

In Fig. 15c, it can be seen that the proposed converter's output voltage varies with different switching frequencies. The new design of the proposed converter enables high output voltage at high switching frequencies with very low values of inductors and capacitors. However, the load voltage of the proposed converter significantly reduces when the switching frequency is reduced to 25 kHz. This means that the proposed converter can supply 400 V to a 400 W load at a duty cycle of 33% by utilizing high switching frequency, resulting in a small size and low cost of the proposed converter. Figure 15d displays the overall power losses of the proposed converter, taking into account various input and output voltages. One significant observation is that the converter experiences a loss of 4% at 400 W when the input voltage is around 40 V. The proposed converter demonstrates remarkable efficiency when supplying high load current at a low duty cycle within the input voltage range of 20–40 V.

**Figure 16:**
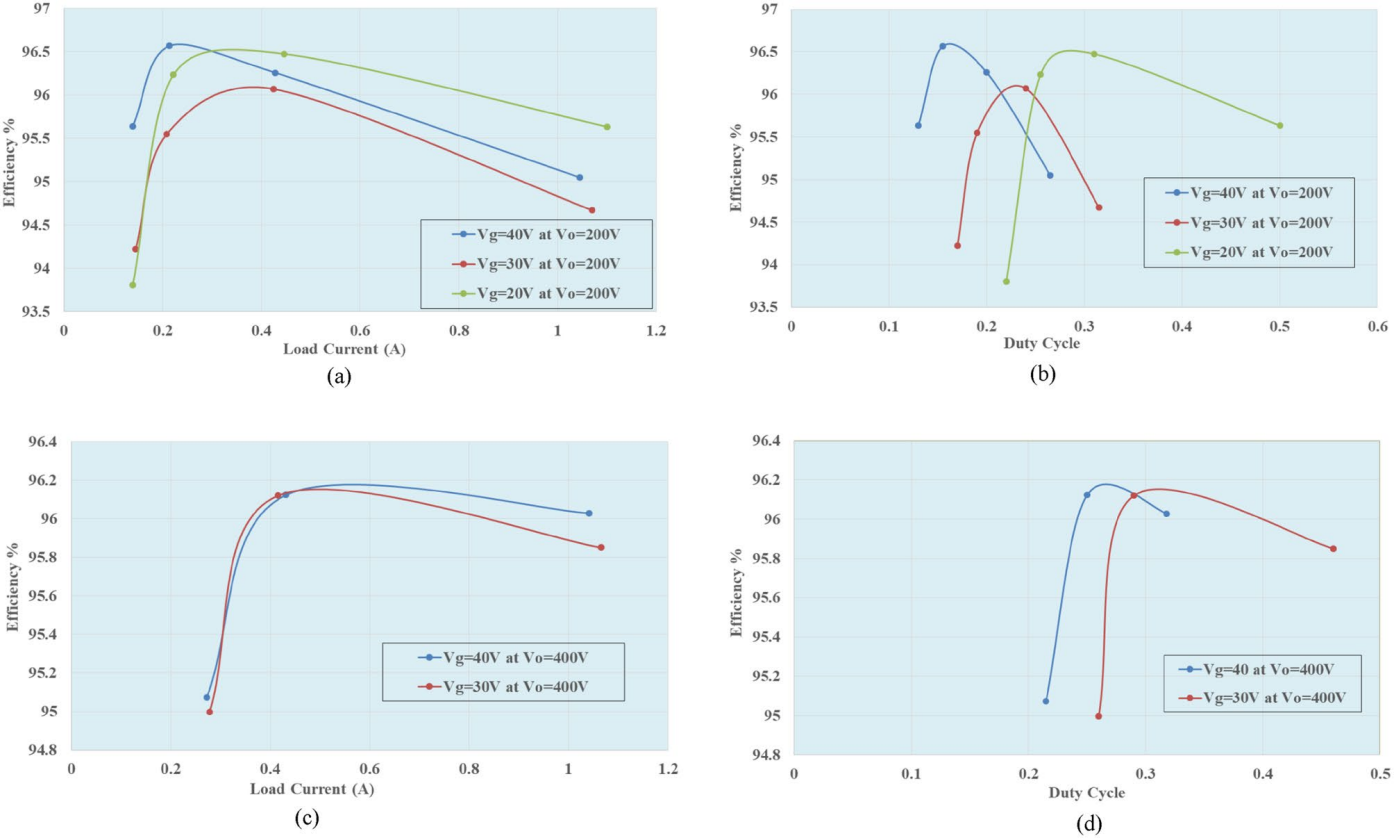
(**a**–**d**) Efficiency of the proposed converter at variable input and output voltage versus load current and duty cycle respectively.

In Fig. 16a,b, the proposed converter's efficiency is shown under variable input voltage at an output voltage 200 V. The results indicate an efficiency of around 96% at 40 V input voltage. In Fig. 16c,d, the efficiency of the proposed converter is shown for variable input voltage at an output voltage of 400 V. The results indicate an efficiency of approximately 96% at 40 V input voltage.

Therefore, the suggested converter is capable of increasing a low input voltage to a high output voltage while providing a high load current at variable input voltage without any input current pulsation at a low duty cycle with high power density efficiency. In addition, the proposed DC–DC converter can supply variable high-output voltage between 200 and 400 V, making it more suitable for a wide range of applications.

## Conclusion

In conclusion, this paper has introduced a highly optimized DC–DC converter employing the innovative modified switched inductor-capacitor (MSLSC) technique, which represents a significant advancement in the field of (RES). The MSLSC-based converter offers several remarkable advantages that make it a promising solution for various applications. One of the key contributions of this research is the introduction of the modified switched inductor (MSL1) and capacitor in series with diodes, which operate in a resonant mode. This design effectively reduces current stress across the main switch, diodes, and inductors, thereby enhancing the overall reliability of the converter. Additionally, the integration of modified switched inductors (MSL2) and capacitors with auxiliary switches further boosts voltage gain and reduces voltage stress on critical components. In addition, the proposed DC–DC converter can supply variable high-output voltage between 200 and 400 V, making it more suitable for a wide range of applications.

The experimental results, including the construction of a 400 W printed circuit board (PCB), validate the simulation findings, demonstrating a remarkable efficiency of approximately 96.2% at 400 W with a 40 V input voltage. This level of efficiency is a significant achievement, especially for RES applications, where converting low input voltages to high output voltages is crucial. Furthermore, the elimination of pulsating input current and reduced voltage stress on power devices highlight the practicality and reliability of the proposed converter. By leveraging SiC MOSFETs and high switching frequencies, this converter minimizes switching losses, component values, and circuit size while maximizing efficiency and performance.

In summary, the optimized DC–DC converter based on the MSLSC technique represents a groundbreaking development in RES. Its exceptional efficiency, voltage stress reduction, and adaptability to various duty cycles make it a promising candidate for enhancing the efficiency and reliability of RES applications. This innovation holds great potential for contributing to the advancement of sustainable energy solutions.

## Data Availability

All data is available upon request.
